# A Multi-Trait, Meta-analysis for Detecting Pleiotropic Polymorphisms for Stature, Fatness and Reproduction in Beef Cattle

**DOI:** 10.1371/journal.pgen.1004198

**Published:** 2014-03-27

**Authors:** Sunduimijid Bolormaa, Jennie E. Pryce, Antonio Reverter, Yuandan Zhang, William Barendse, Kathryn Kemper, Bruce Tier, Keith Savin, Ben J. Hayes, Michael E. Goddard

**Affiliations:** 1 Victorian Department of Environment and Primary Industries, Bundoora, Victoria, Australia; 2 CSIRO Animal, Food and Health Sciences, Queensland Bioscience Precinct, St. Lucia, Queensland, Australia; 3 Animal Genetics and Breeding Unit, University of New England, Armidale, New South Wales, Australia; 4 School of Land and Environment, University of Melbourne, Parkville, Victoria, Australia; The Wellcome Trust Centre for Human Genetics, University of Oxford, United Kingdom

## Abstract

Polymorphisms that affect complex traits or quantitative trait loci (QTL) often affect multiple traits. We describe two novel methods (1) for finding single nucleotide polymorphisms (SNPs) significantly associated with one or more traits using a multi-trait, meta-analysis, and (2) for distinguishing between a single pleiotropic QTL and multiple linked QTL. The meta-analysis uses the effect of each SNP on each of *n* traits, estimated in single trait genome wide association studies (GWAS). These effects are expressed as a vector of signed t-values (**t**) and the error covariance matrix of these t values is approximated by the correlation matrix of t-values among the traits calculated across the SNP (**V**). Consequently, **t'V^−1^t** is approximately distributed as a chi-squared with *n* degrees of freedom. An attractive feature of the meta-analysis is that it uses estimated effects of SNPs from single trait GWAS, so it can be applied to published data where individual records are not available. We demonstrate that the multi-trait method can be used to increase the power (numbers of SNPs validated in an independent population) of GWAS in a beef cattle data set including 10,191 animals genotyped for 729,068 SNPs with 32 traits recorded, including growth and reproduction traits. We can distinguish between a single pleiotropic QTL and multiple linked QTL because multiple SNPs tagging the same QTL show the same pattern of effects across traits. We confirm this finding by demonstrating that when one SNP is included in the statistical model the other SNPs have a non-significant effect. In the beef cattle data set, cluster analysis yielded four groups of QTL with similar patterns of effects across traits within a group. A linear index was used to validate SNPs having effects on multiple traits and to identify additional SNPs belonging to these four groups.

## Author Summary

We describe novel methods for finding significant associations between a genome wide panel of SNPs and multiple complex traits, and further for distinguishing between genes with effects on multiple traits and multiple linked genes affecting different traits. The method uses a meta-analysis based on estimates of SNP effects from independent single trait genome wide association studies (GWAS). The method could therefore be widely used to combine already published GWAS results. The method was applied to 32 traits that describe growth, body composition, feed intake and reproduction in 10,191 beef cattle genotyped for approximately 700,000 SNP. The genes found to be associated with these traits can be arranged into 4 groups that differ in their pattern of effects and hence presumably in their physiological mechanism of action. For instance, one group of genes affects weight and fatness in the opposite direction and can be described as a group of genes affecting mature size, while another group affects weight and fatness in the same direction.

## Introduction

Polymorphisms that affect complex traits (quantitative trait loci or QTL) may affect multiple traits. This pleiotropy is the main cause of the genetic correlations between traits, although another possible cause of genetic correlation is linkage disequilibrium (LD) between the QTL for different traits. A positive genetic correlation that is less than 1.0 between two traits, such as weight and fatness, implies that some QTL affect both traits in the same direction, but other QTL may affect only one trait and a small number may even affect the traits in the opposite direction. Identifying QTL with different patterns of pleiotropy should help us to understand the physiological control of multiple traits. Although genome wide association studies (GWAS) are usually performed one trait at a time, it is not uncommon to find that two traits are associated with SNPs in the same region of a chromosome. This has been described as cross phenotype association [1]. Resolving whether cross phenotype associations are due to one QTL with pleiotropic effects or two linked QTL [1] has proved challenging, given the large number of loci that appear to cause variation in complex traits [2]–[5].

In practice, the apparent effect of a SNP on a trait is estimated with some experimental or sampling error. Consequently, even if there is a single QTL in a region of the chromosome, the SNP with the strongest association may vary from one trait to another causing the estimated position of the QTL to vary between traits. If one QTL can explain the findings for the multiple traits then a multi-trait analysis might result in higher power to detect QTL and greater precision in mapping them. Multiple-trait analysis of linkage experiments has been reported to increase the power to detect QTL [6], [7]. This paper investigates whether additional power can be extracted from a GWAS by analyzing traits together rather than one at a time.

In principle, provided the computing power exists, a multi-trait GWAS is statistically straightforward. However, typically not all subjects have been measured for all traits, and when different traits have been investigated in different experiments, the individual subject data may not even be available. Therefore we present an approximate, multi-trait meta-analysis that uses as data the estimated effects of the SNPs from *n* individual trait GWAS.These effects are expressed as a vector of signed t-values (**t**) and the error covariance matrix of these t values is approximated by a *n*×*n* correlation matrix of t-values among the traits calculated across the SNP (**V**). Consequently, **t'V^−1^t** is approximately distributed as a chi-squared with *n* degrees of freedom. The meta-analysis used here, although approximate, appropriately models the variances and covariance among the t-values regardless of the overlap in individuals measured for the different traits. The different amount of information for the different traits (e.g. different number of individuals genotyped, size of error variance relative to SNP effect) is accounted for in the analysis.

To distinguish between pleiotropy and multiple, linked QTL, we use two different analyses. Firstly, we consider whether all SNPs in a region do or do not show the same pattern of effects across traits. Secondly, we fit the most significant SNP from the multi-trait analysis in the model to test whether this does or does not eliminate the evidence for a second QTL.

An aim of GWAS is to identify the genes and polymorphic sites in the genome that cause variation in complex traits. Choosing the most likely candidate genes from the region surrounding a SNP is usually based on the relationship between the function of the gene and the trait. Assuming that some QTL show pleiotropy, the pattern of pleiotropic effects would be an important clue to the nature of the causative mutation and the function of the gene in which it occurred. Genes that belong to the same pathway might have a similar pattern of pleiotropic effects. Therefore we investigate whether QTL can be clustered into groups with a similar pattern of pleiotropic effects and hence into physiologically similar groups.

The objectives of this study were to test the power of a multi-trait, meta-analysis to detect and map pleiotropic QTL affecting growth, feed conversion efficiency, carcass composition, meat quality and reproduction in beef cattle. We also investigate whether these pleiotropic QTLs can be placed in groups with a similar pattern of effects and hence similar underlying physiological mechanisms.

## Results

### Power of multi-trait meta-analysis to detect QTL

#### False discovery rate

In this study, the 10,191 cattle had real or imputed genotypes for 729,068 SNP, although not all cattle were measured for all traits. The cattle were sourced from 9 different populations (Angus, Murray Grey, Shorthorn, Hereford, Brahman, Belmont Red, Santa Gertrudis, Tropical composites, and recent Brahman crosses). Single trait genome wide association studies were performed for the 32 traits listed in Table 1 and the results have been previously reported by Bolormaa et al. [8]. The traits include measures at different ages of height, weight, fatness, muscularity, feed intake, meat tenderness, age at puberty, and interval of postpartum anoestrus (Table 1).

**Table 1:** Number of records, mean, standard deviation (SD), heritability estimate (h^2^) of each trait for the genotyped animals and their 5-generation ancestors.^1^

Trait ID	No. of Animals	Mean	SD	h^2^	Trait name
PW_hip	6359	120.5	8.1	0.55	Hip height measured post weaning (cm)
SF_hip	1854	131.7	8.1	0.51	Hip height measured at feedlot entry (cm)
X_hip	2037	139.2	8.2	0.36	Hip height measured at feedlot exit (cm)
HUMP	1132	139.7	38	0.34	Hump height as assessed by MSA grader (mm)
PW_lwt	9884	238.9	55.6	0.42	Live weight measured post weaning (kg)
X_lwt	5992	504.2	95.8	0.44	Live weight measured at feedlot exit (kg)
MIDWT	1585	89.5	14.6	0.72	Metabolic mid-test weight in the RFI test period (kg^0.73^)
ADG	1936	1.4	0.4	0.43	Average daily gain over RFI test period (kg)
RFI	4026	−1.4	2.1	0.38	Residual feed intake (kg)
PWIGF	918	276.6	149.3	0.37	IGF-I concentration measured post weaning (ng/ml)
EIGF	1103	510.1	186.6	0.17	IGF-I measured at feedlot entry (ng/ml)
XIGF	948	621	133.6	0.2	IGF-I measured at feedlot exit (ng/ml)
CP8	5727	11.3	4.7	0.39	P8 fat depth at slaughter (mm)
CRIB	5464	7.6	4.1	0.34	rib fat at slaughter (mm)
SP8	4779	8	3.6	0.54	Exit scanned P8 fat depth (mm)
SRIB	4779	11.2	4.5	0.52	Exit scanned rib fat (mm)
CIMF	5824	3.6	2	0.4	Percent intramuscular fat measured in Longissimus lumborum muscle (%)
CMARB	4228	0.8	0.8	0.27	Ausmeat marble score as assessed by MSA grader (score)
CEMA	1557	75.1	8.6	0.43	Eye muscle area at slaughter (cm^2^)
SEMA	4539	68.1	10.9	0.17	Exit scanned eye muscle area (cm^2^)
CRBY	2684	67	3.4	0.46	Carcass retail beef yield (%)
LLPF	5358	4.5	1	0.3	Peak force measured in Longissimus lumborum muscle (kg)
SC12	1112	21.2	2.7	0.62	Scrotal circumference measured at ages of 12 months (cm)
PNS24	964	73.6	22.1	0.23	Percentage of normal sperm at the age of 24 months (%)
AGECL_BB	1007	751	114.9	0.56	Age at first detected corpus luteum in BB (days)
AGECL_TC	1108	650	104.8	0.49	Age at first detected corpus luteum in TC (days)
PPAI_BB	629	180	3.8	0.51	Post partum anoestrus interval in BB (days),
PPAI_TC	863	141	3	0.29	Post partum anoestrus interval in TC (days),
WTCL_BB	993	334	42	0.56	Live weight measured at the age when the first corpus luteum in BB (kg)
WTCL_TC	1094	329	41.3	0.46	Live weight measured at the age when the first corpus luteum in TC (kg)
P8CL_BB	951	4.5	2.1	0.47	P8 fat depth measured at the age when the first corpus luteum in BB (mm)
P8CL_TC	1083	3	1.5	0.42	P8 fat depth measured at the age when the first corpus luteum in TC (mm)

1 = similar summary statistics for 19 of the above traits can be found in [8].

In the multi-trait analysis we measured the effect of a SNP on each trait by a signed t-value (effect/standard error of effect) and approximated the (co)variance matrix among the traits using the correlation matrix of these SNP effects. Then the effects of a SNP across the 32 traits were combined with this correlation matrix to perform a multi-trait chi-squared test with 32 degrees of freedom of the null hypothesis that a SNP has no effect on any trait.

**Figure 1:**
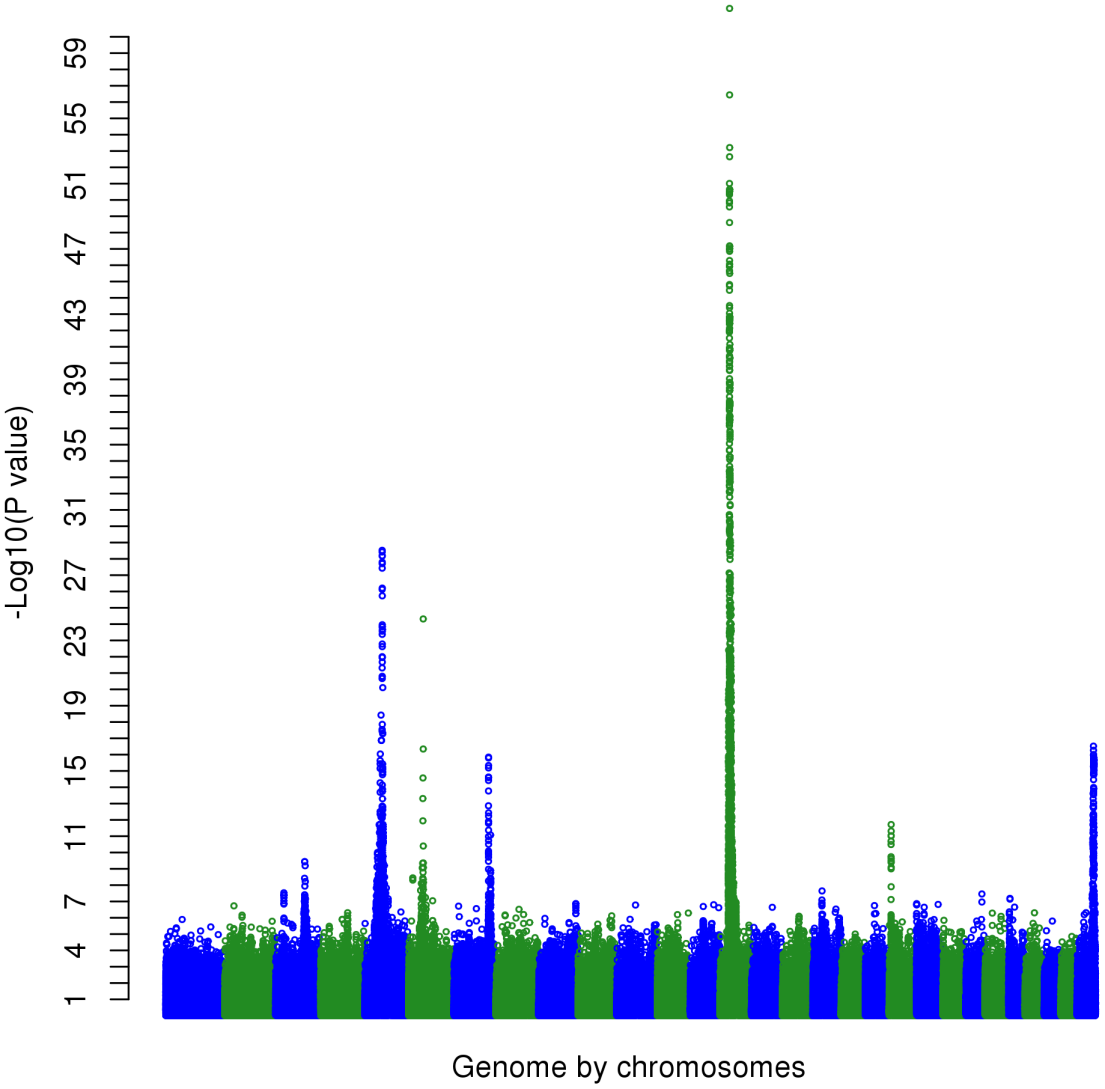
The Manhattan plot showing the −log_10_(*P*-values) of SNPs of the multi-trait test of the whole genome except the X chromosome.

For this test, 2,028 SNPs were significant (*P*<5×10^−7^) (Figure 1). This corresponds to a false discovery rate (FDR) of 0.02%, and this was lower than for any individual trait; when traits were analyzed individually, for 19 out of 32 traits the FDR at *P*<5×10^−7^ ranged from 0.04% to 2.5% and for the 13 remaining traits FDR was greater than 3.5% (Table 2). Therefore the multi-trait test had greater power to detect QTL than the individual trait analyses.

**Table 2:** Number of SNPs and their false discovery rates (%) at *P*<5×10^−7^ for each trait before and after fitting the 28 leading SNPs in the model.

	Without 28 lead SNPs		With 28 lead SNPs	
Trait*	No.	FDR	No.	FDR
PW_hip	912	0.0	62	0.6
SF_hip	36	1.0	28	1.2
X_hip	82	0.4	2	17.3
HUMP	14	2.5	14	2.5
PW_lwt	545	0.1	32	1.1
X_lwt	543	0.1	8	4.3
MIDWT	4	8.7	1	34.6
ADG	17	2.0	1	34.6
RFI	25	1.4	12	2.9
PWIGF	267	0.1	1	34.6
EIGF	280	0.1	1	34.6
XIGF	5	6.9	5	6.9
CP8	325	0.1	6	5.8
SP8	24	1.4	6	5.8
CRIB	36	1.0	4	8.7
SRIB	0		0	
CIMF	58	0.6	10	3.5
CMARB	1	34.6	1	34.6
CEMA	0		0	
SEMA	0		0	
CRBY	36	1.0	7	4.9
LLPF	547	0.1	8	4.3
SC12	3	11.5		
PNS24	1	34.6		
AGECL_BB	497	0.1		
AGECL_TC	0			
PPAI_BB	2	17.3		
PPAI_TC	10	3.5		
WTCL_BB	406	0.1		
WTCL_TC	180	0.2		
P8CL_BB	0			
P8CL_TC	0			

*  = empty cells are not available.

#### Validation of SNP effects

To validate the associations found in the multi-trait analysis, we used individual level data. The data were split into a discovery sample and a validation sample. The whole data were split into five sets by allocating all of the offspring of randomly selected sires to one of the five datasets. Then one of the 5 divisions was randomly selected as a validation population and the other 4 divisions as the reference population. In this way no animal used for validation had paternal half sibs in the reference population.

From the multi-trait analysis of the discovery dataset, the most significant SNPs (*P*<10^−5^) were retained and then to avoid identifying a large number of closely linked SNPs whose association with traits is due to the same QTL, only the most significant SNP in a 1 Mb interval was selected for validation.

For each SNP we calculated the linear index of 22 traits. This linear index had maximum correlation with the corresponding SNP. Then the association between a SNP and its corresponding linear index was tested in the validation sample. To do this we needed individual animals that had been measured for nearly all traits. However, the bulls and cows, which had been measured for 10 reproductive traits, were not measured for the other 22 traits. Therefore we based the validation on animals measured for the 22 non-reproductive traits and calculated the linear index for each SNP based on these 22 traits (Table 3). Out of the 244 significant SNPs, 207 or 85% had an effect in the same direction in the validation sample as in the discovery sample. The size of the validation sample (1,899 animals) limited its power but 72 of the 244 SNPs were significant (*P*<0.05) and 71 of the 72 had an effect in the same direction as in the discovery sample.

**Table 3:** Number of significant SNPs (*P*<10^−5^) in reference population that were also significant in the validation population.

*P*_value in validation	No. of SNP	FDR%	%-same
*multi-trait*			
0.0001	5	0.5	100
0.001	9	2.6	100
0.01	35	6.0	97
0.05	72	12.6	99
all	244		85
*single-trait* (PW_lwt)			
0.0001	0		
0.001	0		
0.01	8	9.0	100
0.05	13	26.7	100
all	79		76

%-same = percentage of SNPs, which have an effect in the same direction in both validation and reference sets.

To compare the power of detecting QTL in the multi-trait analysis to that in the single-trait analysis, we performed the same validation analysis for the single trait post weaning live weight (PW_lwt), which is one of the traits with the highest number of significant associations (Table 3). For PW_lwt, only 79 SNP met the criterion (*P*<10^−5^ and one SNP per Mb) and of these 60 (76%) had an effect in the same direction in the validation sample as in the discovery sample, but only 13 of the 79 were significant at *P*<0.05 in the validation sample. This shows that multi-trait analysis detected more associations and validated a higher percentage of them than the best single trait analysis. For other single traits the proportion of significant SNPs from the reference that were validated was even lower than for PW_lwt.

### Precision of multi-trait meta-analysis for QTL mapping

**Figure 2:**
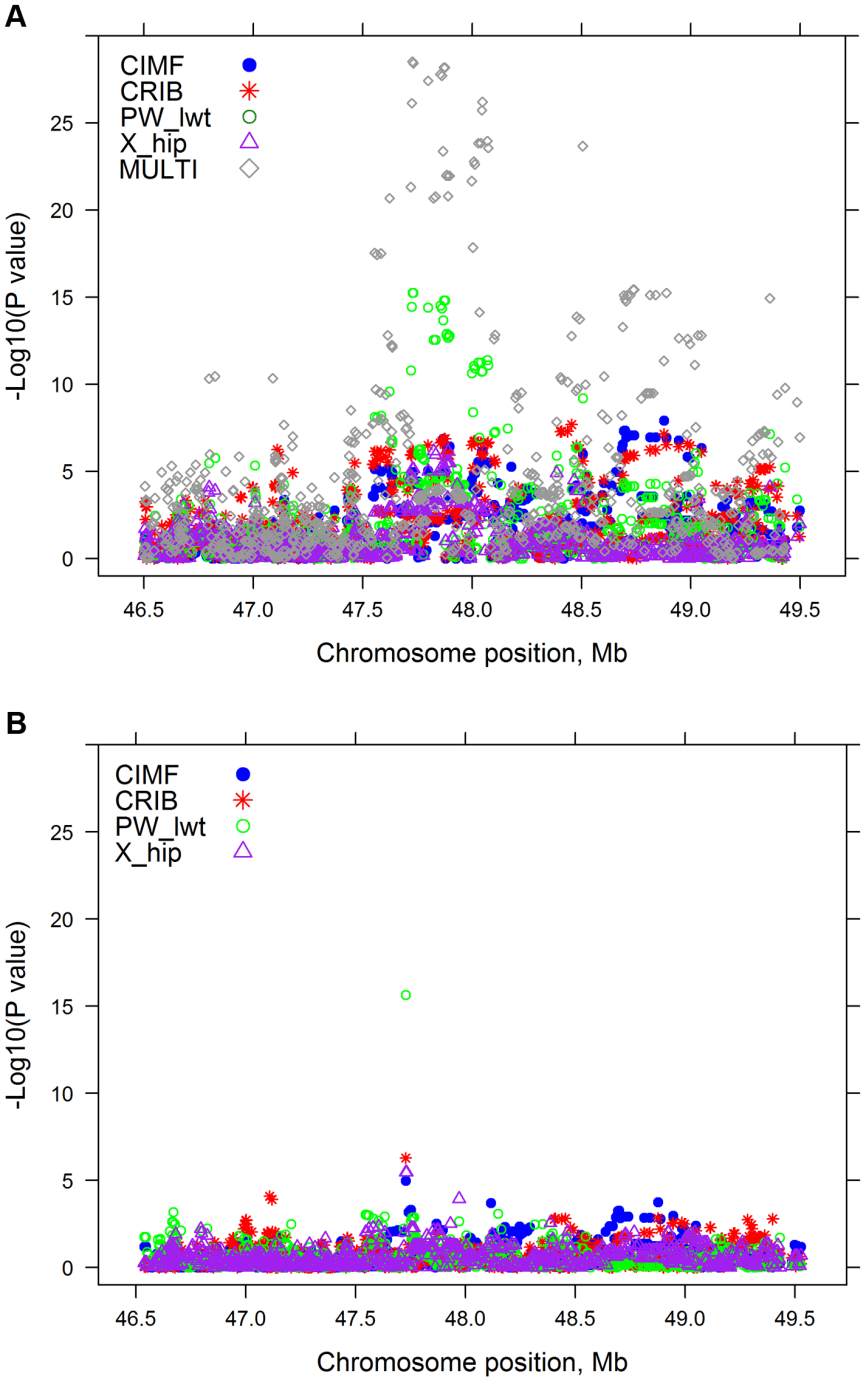
A: The −log_10_(*P*-values) of single SNP regressions for 4 traits and multi-trait chi-squared statistic on a region of BTA 5; B: The −log_10_(*P*-values) of single SNP regressions for 4 traits when SNP_i_ along with 28 lead SNPs were simultaneously fitted in the GWAS model.

Many of the significant SNPs in both single trait and multi-trait analyses were linked and might be associated with the same QTL. As an example of the multi-trait approach to improve precision, Figure 2A shows the significance of SNP effects for 4 single trait GWAS and our multi-trait statistic in a region of chromosome 5 (BTA 5). The 4 separate traits map the QTL to slightly different positions (range: 47,732–48,877 kb). For the multi-trait statistic, based on SNP effects from single-trait GWAS for 32 traits, the most significant SNP (*P* = 1.32×10^−27^) was located at 47,728 kb. The multi-trait analysis represents a good compromise between the positions from the 4 single trait GWAS and may be the best guide to a single QTL position explaining all the associated traits.

### Multi-trait meta-analysis tends to find SNPs near genes

**Figure 3:**
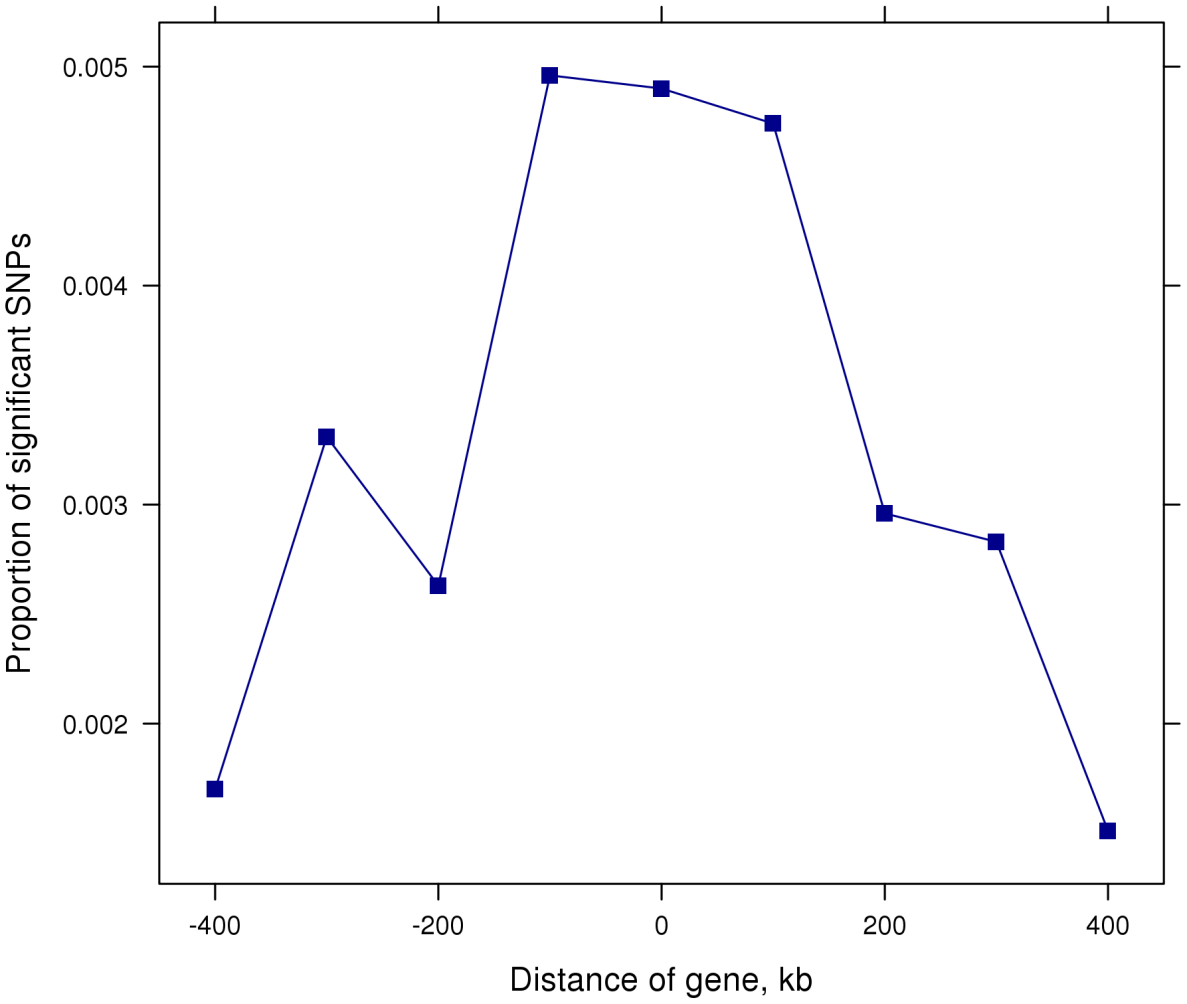
Proportion of significant (P<10^−5^) SNPs in 100 kb steps from gene start and stop positions. Position = 0 indicates SNPs between start and stop positions.

SNPs were classified according to their distance from the nearest gene and the proportion of SNPs at each distance from a gene that were significant (*P*<10^−5^) in the multi-trait analysis was calculated. Figure 3 shows that SNPs were more likely to be significantly associated with the 32 traits if they were within or less than 100 kb from a gene.

### Single-trait GWAS to test pleiotropy or linkage

**Figure 4:**
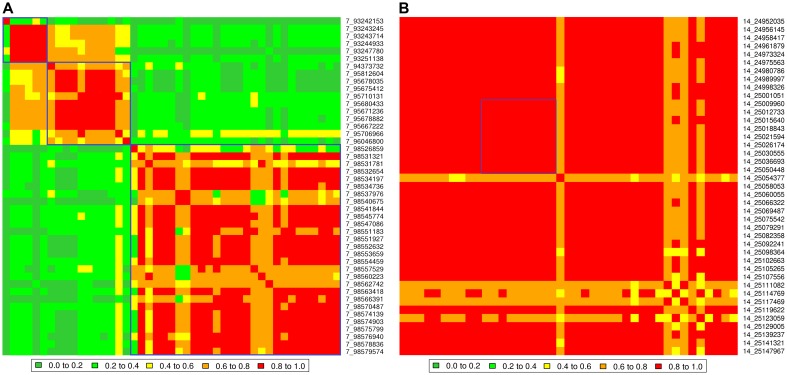
Correlations between pairs of the SNP effects on 32 traits. A: Correlations on BTA7 from 93 Mb to 99 Mb. Three blocks of SNPs with high correlations within a block and low correlation between blocks are shown in blue. B: Correlations on BTA 14 near 25 Mb. The blue line shows the SNPs closest to the *PLAG1* gene.

There are many regions of the genome, similar to that illustrated in Figure 2A, where multiple traits had significant associations with one or more SNPs. For each SNP their estimated effects on each trait were expressed as a signed t-value. For each pair of SNPs we calculated the correlation across the 32 traits between their estimated effects so that SNPs with the same pattern of effects across traits are highly positively or negatively correlated. Figure 4 shows the correlation between SNPs on a region of chromosomes 7 and 14. All SNPs in the vicinity of 25 Mb on chromosome 14 are highly correlated indicating a single pleiotropic QTL in this region, corresponding to previous reports of a polymorphism near the gene *PLAG1* that affects many traits [9]–[11]. On chromosome 7 there are three blocks of SNPs with high correlations within a block and low correlations between blocks suggesting there are three QTL, close to 93, 95 and 98 Mb. The QTL at 98 Mb corresponds to a previously reported polymorphism in calpastatin (*CAST*) [12], [13]. Below, we confirm this interpretation by fitting the most significant SNPs in the model and testing for additional associations.

### Conditional analyses to test pleiotropy or linkage

#### Detection of pleiotropic QTL

**Figure 5:**
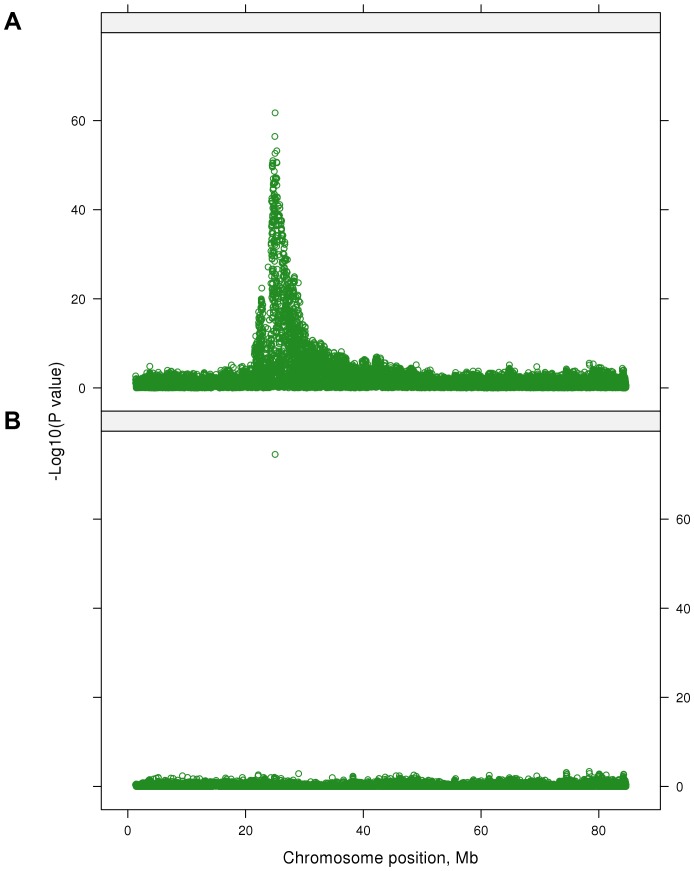
The −log_10_(*P*-values) of the multi-trait test calculated using SNP effects from the single-trait GWAS for 32 traits on BTA 14 before (A) and after (B) fitting 28 lead SNPs in the model. In (B) the significance of the lead SNP is also given after fitting the other 27 lead SNPs.

Many highly significant SNPs from the multi-trait analyses were found within narrow regions on chromosomes (BTA) 3, 5, 6, 7, 14, 20 and 29 (Figure 1). If there is only a single QTL in a region and if it is perfectly tagged by one of the SNPs, then when this SNP is fitted in the model the other nearby SNPs should have no significant association with the phenotypes. To test this hypothesis we selected 28 ‘lead’ SNPs (Table 4), representing what appeared to be 28 QTL across the genome. GWAS were re-performed but the SNP_i_ (SNP_i_, i = 1, 2, 3, …, 729068) along with the 28 lead SNPs were simultaneously fitted in the model, and then the multi-trait statistic was re-calculated for SNP_i_ to test the effects of the SNP_i_ across traits after fitting the 28 lead SNPs. Figure 5 shows the results for BTA 14 as an example. In the original multi-trait GWAS, many SNPs between 20 and 40 Mb on BTA 14 were significant but after fitting the 28 lead SNPs, which include one at 25 Mb on BTA 14, there were no more significant SNPs in this region than in the rest of BTA 14 (Figure 5 shows the lead SNP as well as all other SNPs).

**Table 4:** Description of the 28 lead SNPs and their *P* values in the genome wide association studies (GWAS).

Group^1^	SNP order	SNP name	BTA^2^	Position	*P* value	Linear Index^3^
1	1	BovineHD2000001543	20	4873556	1.1E-16	BTA20_4.9 Mb
1	2	BovineHD1400007259	14	25015640	1.1E-16	BTA14_25 Mb
1	3	BovineHD0500013788	5	47727773	1.1E-16	BTA5_47.7 Mb
1	4	BovineHD0600010976	6	40093712	1.1E-16	BTA6_40.1 Mb
2	5	BovineHD2600007456	26	28012143	2.4E-08	BTA26_28.0 Mb
2	6	BovineHD2500000802	25	3747518	2.0E-06	BTA25_3.7 Mb
2	7	BovineHD1000026655	10	92188144	7.9E-08	BTA10_92.2 Mb
2	8	BovineHD0700028765	7	98540675	1.1E-16	BTA7_98.5 Mb
2	9	BovineHD2900015063	29	44837096	1.1E-16	BTA29_44.8 Mb
3	10	BovineHD0300023058	3	80105316	3.2E-15	BTA3_80.1 Mb
3	11	BovineHD1700007012	17	24884021	2.7E-09	BTA17_24.9 Mb
3	12	Hapmap42512-BTA-32321	13	34909187	5.0E-11	BTA13_34.9 Mb
3	13	BovineHD0200007253	2	25222940	5.9E-06	BTA2_25.2 Mb
3	14	BovineHD2500004094	25	14547288	1.7E-11	BTA25_14.5 Mb
3	15	BovineHD0600003225	6	12748745	1.3E-08	BTA6_12.7 Mb
3	16	BovineHD1900007319	19	25052604	2.3E-08	BTA19_25.1 Mb
4	17	BovineHD1700017438	17	61227950	2.0E-10	BTA17_61.2 Mb
4	18	BovineHD0800017674	8	59156184	3.3E-08	BTA8_59.2 Mb
4	19	BovineHD0400021462	4	77561148	8.6E-11	BTA4_77.6 Mb
4	20	BovineHD1300018707	13	65917704	3.1E-07	BTA13_65.9 Mb
4	21	BovineHD1200013180	12	47984330	6.9E-11	BTA12_48.0 Mb
4	22	BovineHD0900004919	9	18195454	8.6E-07	BTA9_18.2 Mb
4	23	BovineHD1500016882	15	58463005	6.3E-10	BTA15_58.5 Mb
4	24	BovineHD2300012740	23	43919433	8.9E-11	BTA23_43.9 Mb
4	25	BovineHD2100000105	21	898385	1.6E-05	BTA21_0.9 Mb
4	26	BovineHD2100005354	21	19018980	7.1E-11	BTA21_19.0 Mb
4	27	BovineHD0900029140	9	100532649	2.2E-09	BTA9_100.5 Mb
4	28	BovineHD0700027239	7	93244933	1.1E-16	BTA7_93.2 Mb

1 = Group of the lead SNPs that were clustered together as shown on Figure 7.

2 = *Bos taurus* chromosome number.

3 = 28 linear indexes corresponding to the 28 lead SNPs.

**Figure 6:**
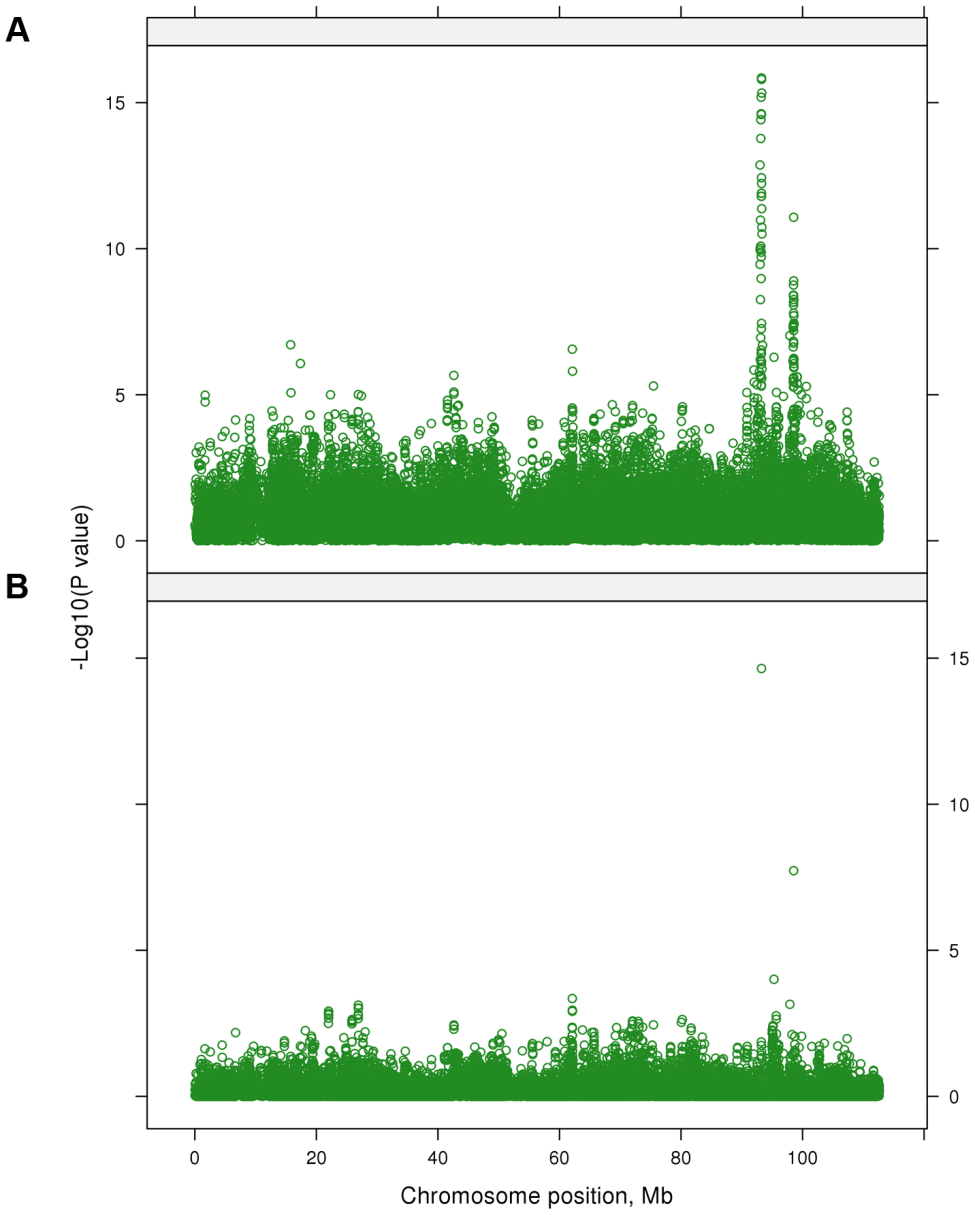
The −log_10_(*P*-values) of the multi-trait test calculated using SNP effects from the single-trait GWAS for 32 traits on BTA 7 before (A) and after (B) fitting 28 lead SNPs in the model. In (B) the significance of the lead SNP is also given after fitting the other 27 lead SNPs.

All 28 lead SNPs remained significant in this conditional analysis, even after fitting the other 27, showing that each tags a different QTL. For instance, on BTA 7 the 2 lead SNPs at 93 and 98 Mb remain significant as does a SNP at 95 Mb (Figure 6). This confirms the interpretation of the correlation analysis (Figure 4) that there are 3 QTL in this narrow region. The apparent effects of the 28 lead SNPs on the 32 traits, as estimated in the original single-trait GWAS, are given in Table 5 (only values with |t|>1 are reported).

**Table 5:** Effects of 28 lead SNPs on the individual traits (signed values with |t|>1 are shown).

	Group 1*				Group 2*					Group 3*							Group 4*											
SNP order^2^	1	2	3	4	5	6	7	8	9	10	11	12	13	14	15	16	17	18	19	20	21	22	23	24	25	26	27	28
PW_hip		10.9	9.6	9.5	−1.5						−1.9				1.9		1.8		3.0	2.0				1.5	−1.5	−4.3		−2.0
SF_hip	1.6	3.7	3.9	5.8		−2.1						−1.1	−1.8	−3.1		−2.8	1.4	1.0				1.7	1.8			−3.4		
X_hip		6.3	4.5	5.5		−1.5	−1.5	−1.8		1.5				−3.6		−1.3		−1.6	−1.4						−1.1	−1.8		
HUMP				1.1	2.1	4.4			−1.2		−1.3				−2.4		1.2		−4			−1.5	1.1	1.2			1	1.4
PW_lwt	4.4	9.8	8.1	8.1	−4.0		−1.6	1.7	−1.1	1.6			−2.0	−1.9			3.2	−1.4	2.1			2.5	2.7	−2.1		−1.7		2.3
X_lwt	6.4	10.2	6.1	3.7		−1.0	−2.2			6.1	2.2	2.3			1.2	1.2	3				1.6	−1.1					2.6	3.7
MIDWT	3.3	5.1	2.7				2.0	2.0		3.5		3.3	2.5	1.2	3.2	4.1			−1.3		2.8	−1.1	1.9	−1.3		−2.7		2
ADG	2.1	2.1	1.5				1.3	1.2		5.0	4.9	3.3	2.0		2.1	3.1	−1.5		−1.7		1.4							2.2
RFI	−1.2	−4.7	−2.8	−2.2		1.5	1.2				−1.8	2		3.8	3.1	2.1	2.6	1.6		−1.9			−3.1		−1.7	−2.0	−2.2	−3.5
PWIGF	−3.2	−7.6	−1.9	−2.6		−1.9	2.3	2.1			−1.3	−1.2		−4.0	3.7		1.8					−1.7				−1.4	−1.7	−2
EIGF	−2.0	−7.1	−3.9	−4	−1.1		1.7				−1.4		1.3	1.8	1.5	1.1	−1.3	1.3			1.7		3.6	−2.2	1.7			−1.6
XIGF	−1.2	−3.5	−2.0	−1.3			2.1		1.3	1.4	1.1	−2.2					2	3.2	1.6		2.3				2.4		−1.9	−1.6
CP8	−1.2	−7.0		−1.9		1.3	1.4	1.1	3.6	1.1					1.4		−2.1	−3.3	−2.5		2.5	−3.5					2	
SP8	−1.4	−4.3		−1.1	−2.4	2.7		1.6	2.3	3.8	2.3		3.9	1.3	2.0	1.6	−1.8		−1.4	2.5	−1.1		3	−2.6			−1.7	−2.1
CRIB	−1.1	−4.1	−5.0	−2.6		1.3	1.4	1.4	2.3	3.0	1.7			1.2	3.6		−2.4	−1.3			−1.2			−2.0			−2.8	
SRIB	−1.4	−3.7	−2.7		−1.1			2.0	2.4	4.2	2.7	2.9	3.7	2.2	1.3		−1.6	−2.2	−2.3				1.9	−3.5	−1.8		−1.3	−2.6
CIMF	−2.7	−1.6	−4.4	−2.5	2.6	2.6	1.9	1.5	3.7	2.5	−1.8	−1.4		2.5	1.9	2.9		1.7	1.7				−3	−1.2				−3.5
CMARB	−2.8	−1.7	−2.1			1.5				1.4	−1.6					1.7	−2.5				1.3		−2.2	−1.7	−1.6	−1.4		−2.3
CEMA		−1.0	−3.4	1.7		−2.1	−1.7	1.0		−2.2	−1.5		1.6	1.1	−1.4	−2.3			−1.2		3.1			1.8	1.8	1.5		2.6
SEMA		2.1				−2.1	−2.2		−1.4	3.6			1.0	1.9		1.0		1.3		2.7	2.4	−1.8					1.8	3.7
CRBY	1.6		−2.0		−2.3					−2.0		1.8	−2.1		−1.9	−1.6		1.9	2.1		−1.9			3.4	2.9	3.6	1.4	6.7
LLPF		−2.3	1.9	1.7	−2.9	−2.7	−3.4	−8.6	−10.2				−1.4					−2.9	−2.7	1.5			−1.4	−2.2		−1.9	−1.9	−2.3
SC12	1.1	−4.5	−1.9			2.3	−2.5			2.2		−1.1			−2.5	−1.4	1.0		2.1	2.4		2.3			2.1	1.1		
PNS24	1.1	−1.3	−1.0	−2.4						1.5	2.0	2.5		−1.1				−2.0			1.4	2.5	−1.6			−1.6	1.2	
AGECL_BB	2.5	6.3	1.2	2.0	2.3		1.1						−3.1	−1.7	−3.3			−2.4		3.3		1.1			−1.6			
AGECL_TC	1.2	3.5	3.3	1.1			−1.4				1.0	−1.7			−2.2	−3.1	2.3	1.3		−1.8	1.2	1.2			−4.6		1.3	1.6
PPAI_BB		2.4					−1.3		−2.7		2.9	2.2			−3.2			−2.0	−1.7			−1.6	−1.4		3.2	1.4	−3.4	
PPAI_TC	1.8	2.1	4.3		−2.1	−1.9			−2.3	2.3	1.3	−2.6			−1.2				2.3	−3.6		2.1				1.2		1.3
WTCL_BB	1.4	6.6	1.9	5.3					−1.0				−3.4	−2.7	−3.4		−1	−3.6		2.6	1.1				−2.0		−1.6	
WTCL_TC	3.0	7.0	4.6	1.1		−1.7	−1.7				−1.2	−1.7	1.2	−1.0	−1.1	−3.7	2.1			−2.2		1.5		−2.4	−4.0		1.8	1.1
P8CL_BB	2.3	−2.6	1.4	−2.0	1.4		−1.2			−1.7	1.4	1.2	−3.1					−1.5		2.1	−1.0	1.1	1.1	1.5	−1.3		1.8	−1.6
P8CL_TC	1.1	−2.4		1.2			−2.6			−1.5	−1.2		2.8				−1.2	−1.8	−2.7		1.4				−3.4	−1.3	1.0	

**Figure 7:**
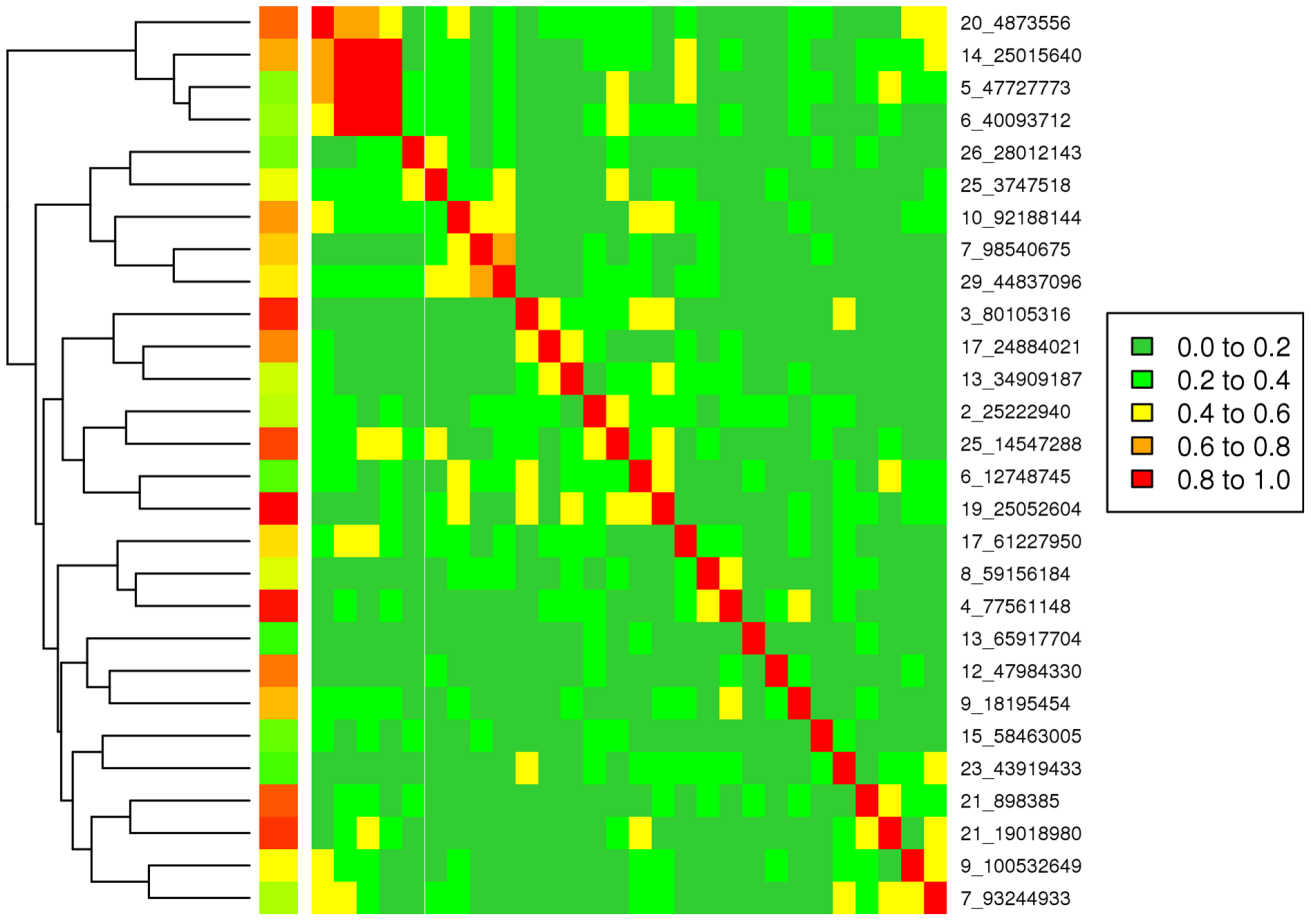
Correlation matrix between the 28 lead SNPs calculated from SNP effects on 32 traits (reordered for constructing a dendrogram).

*  = Group of the lead SNPs that were clustered together as shown on Figure 7.

2 = This SNP order refers SNPs, which are given on Table 4.

In some cases, a SNP close to the lead SNP remains significant even after fitting the 28 lead SNPs. This could be because of imperfect LD between the lead SNP and the causal mutation so that other SNP may explain some of the variance caused by the causal mutation in addition to the lead SNP. Alternatively, there may be more than one causal variant in the same gene each tracked by a different SNP. In fact, there were still many significant SNPs (*P*<5×10^−7^) scattered throughout the genome (eg., there were 62 significant SNPs for PW_hip; Table 2) indicating that there are likely to be many more than 28 QTL affecting these 32 traits.

#### Mapping of pleiotropic QTL

In Figure 2B the significance of SNP effects for 4 single trait GWAS in a region of chromosome 5 is presented, when the *i*th SNP (SNP_i_, i = 1,2,3, …, 729068) along with 28 lead SNPs were simultaneously fitted in the GWAS model. The results from this conditional analysis show that the lead SNP is significant (*P*<10^−5^) for all 4 traits, but once this SNP is included in the model, no other nearby SNPs reach this level of significance for any of the 4 traits.

### Clustering of QTL with similar pattern of effects across traits

For each pair of SNPs among the 28 lead SNPs, the correlation of their effects across the 32 traits was calculated (Figure 7). There are a few correlations with high absolute value, such as between the lead SNPs on BTA 5, 6 and 14, but most correlations are low. A low correlation suggests QTL with different patterns of effects across traits, however sampling errors in estimating SNP effects also reduce the absolute value of the correlation. If two QTL affect the same physiological pathway one might expect them to have the same pattern of effects and hence a high correlation. Cluster analysis based on effects of the SNPs across traits divided the 28 lead SNPs into 4 loosely defined groups (Figure 7), which share patterns of effects across traits (although there are still differences within each group in the exact pattern of effects across traits) (Table 5).

Group 1 consists of 4 lead SNPs located on BTA 5 (BTA5_47.7 Mb), 6 (BTA6_40.1 Mb), 14 (BTA14_25.0 Mb) and 20 (BTA20_4.9 Mb). This group clustered as an outer branch separate from the other 24 lead SNPs (Figure 7), indicating that this group of SNPs clusters more tightly than the other groups. Three of these 4 SNPs were highly correlated amongst each other while the SNP on BTA 20 had slightly lower correlations to the other 3 SNPs. Table 5 shows that these 4 SNPs have an allele that increases height and weight and decreases fatness, RFI and blood concentration of IGF1. They could be described as changing mature size.

Group 2 consists of SNPs on BTA 7 (BTA7_98 Mb), 10 (BTA10_92 Mb), 25 (BTA25_3.7 Mb), 26 (BTA26_28.0 Mb), and 29 (BTA29_44.8 Mb) with high correlations between 2 SNP on BTA 7 and 29. These SNPs have an allele that increases meat tenderness (i.e., decrease shear force) and fatness (i.e., marbling or intra-muscular fat) (Table **5**). The SNPs at BTA7_98.5 Mb and BTA29_45.8 Mb have a large effect on shear force and map to the positions of known genes affecting this trait (Calpastatin and Calpain 1) [12], [14], [15].

Group 3 consists of 7 SNPs that are located on BTA 2 (BTA2_25.2 Mb), 3 (BTA3_80.1 Mb), 6 (BTA6_12.7 Mb), 13 (BTA13_34.9 Mb), 17 (BTA17_24.9 Mb), 19 (BTA19_25.1 Mb), and 25 (BTA25_14.5 Mb). There was weaker clustering and lower correlations between these SNP compared to groups 1 and 2. The SNPs of Group 3 have an allele that increases both fatness and weight but has little effect on height or IGF1 (Table 5). This distinguishes these SNPs from those in Group 1 where the allele that increases weight also decreases fatness and IGF1.

Group 4, the biggest group, consists of 12 SNPs in a loose cluster. Moderate correlations appeared between some SNPs on BTA 7 (BTA7_93.2 Mb), 9 (BTA9_100.5 Mb), 21 (BTA21_0.9 Mb), 21 (BTA21_19.0 Mb), 23 (BTA23_43.9 Mb), 4 (BTA4_77.6 Mb) and 8 (BTA8_59.2 Mb) (Figure 7). This group has an allele that tends to increase muscling, retail beef yield (RBY), tenderness and feed efficiency, and decrease fatness. The clustering did separate the 2 SNPs on BTA 7 with the SNP near 98 Mb belonging to Group 2 and the SNP near 93 Mb belonging to Group 4.

Although the SNPs within a group share some features they also differ in some of their associations. For instance, in Group 1 the SNP on BTA 14 near *PLAG1* has a more marked effect on age at puberty (AGECL) than others in the group; the SNP on BTA5 changes the distribution of fat between the P8 site on the rump and rib site and the intramuscular depot. Thus it is possible for the each SNP to have a unique pattern of associations with phenotypic traits.

### Finding additional QTL in the same pathway

The pattern of pleiotropic effects might be an important clue to the nature of the causative mutation and the function of the gene in which it occurred. Genes that operate in the same pathway might be expected to show the same pattern of pleiotropic effects. For each of the 28 lead SNPs, we searched for additional SNPs with a similar pattern of effects. To do this we used the linear index of 22 traits that showed the highest association with a lead SNP, as previously defined for validation of the multi-trait analysis, and performed a new GWAS using the linear index as a new trait. Table 6 shows the number of significant (*P*<10^−5^) SNPs for the 28 linear indexes corresponding to the 28 lead SNPs. Out of 28 linear indexes, 19 had more than 70 significant SNPs and hence a FDR of less than 10%. Linear index BTA5_47.7 Mb, BTA6_40.1 Mb, BTA14_25.0 Mb, and BTA20_4.9 Mb (where the name corresponds to the location of the QTL defining the pattern of effects) have associations with over 1,000 significant SNPs across the genome. For the index based on the lead SNP BTA5_47.7 Mb, the significant SNPs included 615 SNPs on BTA 5, 64 on BTA 6, 24 on BTA 11, 907 on BTA 14, 19 on BTA 17, 18 on BTA 20. This reiterates the result obtained in the cluster analysis because SNPs on BTA 5, 6, 14 and 20 are the lead SNPs in Group 1 and the additional SNPs on these chromosomes may be tagging the same QTL as the lead SNPs. However, there are also significant SNPs associated with this linear index on BTA 11, 17, 19, 21 and 25.

**Table 6:** Total number of significant SNPs (*P*<10^−5^), their FDR (%), and number of significant SNP on each chromosome (which is in parenthesis) for the 28 linear indexes corresponding to the 28 lead SNPs.

Group^1^	SNP^2^ order	Linear Index^3^	Total No. sig. SNP^4^	FDR (%)	Number of significant SNPs^5^ (chromosome number)
1	1	BTA20_4.9 Mb	1313	0.5	69 (**4**), 21 (**5**), 40 (**6**), 60 (**7**), 8 (**10**), 1036 (**14**), 6 (**18**), 57 (**20**)
1	2	BTA14_25 Mb	2371	0.3	6 (**4**), 180 (**5**), 159 (**6**), 9 (**8**), 1962 (**14**), 8 (**17**), 20 (**20**), 5 (**21**)
1	3	BTA5_47.7 Mb	1668	0.4	615 (**5**), 64 (**6**), 24 (**11**), 907 (**14**), 19 (**17**), 18 (**20**), 5 (**21**), 5 (**25**)
1	4	BTA6_40.1 Mb	1552	0.4	6 (**4**), 184 (**5**), 143 (**6**), 5 (**11**), 1154 (**14**), 13 (**18**), 18 (**20**), 6 (**21**), 6 (**25**)
2	5	BTA26_28.0 Mb	47	14.7	8 (**6**), 8 (**14**), 11 (**18**), 5 (**20**), 6 (**26**)
2	6	BTA25_3.7 Mb	50	13.8	9 (**9**), 7 (**13**), 10 (**21**), 13 (**29**)
2	7	BTA10_92.2 Mb	378	1.8	27 (**7**), 6 (**10**), 127 (**14**), 204 (**29**)
2	8	BTA7_98.5 Mb	343	2.0	64 (**7**), 264 (**29**)
2	9	BTA29_44.8 Mb	559	1.2	5 (**1**), 61 (**7**), 25 (**10**), 458 (**29**)
3	10	BTA3_80.1 Mb	175	4.0	5 (**1**), 130 (**3**), 19 (**13**), 5 (**21**)
3	11	BTA17_24.9 Mb	173	4.0	23 (**1**), 63 (**3**), 11 (**7**), 11 (**13**), 53 (**17**)
3	12	BTA13_34.9 Mb	131	5.3	109 (**13**), 6 (**21**)
3	13	BTA2_25.2 Mb	27	25.6	9 (**9**)
3	14	BTA25_14.5 Mb	26	26.6	8 (**5**), 6 (**6**)
3	15	BTA6_12.7 Mb	70	9.9	20 (**1**), 21 (**7**), 10 (**17**)
3	16	BTA19_25.1 Mb	71	9.8	41 (**7**), 11 (**10**), 10 (**19**)
4	17	BTA17_61.2 Mb	667	1.0	5 (**5**), 18 (**6**), 6 (**8**), 595 (**14**), 6 (**17**), 20 (**20**), 5 (**25**)
4	18	BTA8_59.2 Mb	47	14.7	22 (**8**), 12 (**16**)
4	19	BTA4_77.6 Mb	723	1.0	38 (**4**), 7 (**6**), 667 (**14**)
4	20	BTA13_65.9 Mb	41	16.9	5 (**4**), 7 (**5**), 5 (**13**), 5 (**18**), 6 (**21**),
4	21	BTA12_48.0 Mb	12	57.7	
4	22	BTA9_18.2 Mb	310	2.2	8 (**7**), 294 (**14**)
4	23	BTA15_58.5 Mb	93	7.4	7 (**6**), 45 (**7**), 15 (**10**), 8 (**14**), 5 (**15**)
4	24	BTA23_43.9 Mb	51	13.6	10 (**7**), 11 (**20**), 12 (**22**), 6 (**23**)
4	25	BTA21_0.9 Mb	75	9.2	20 (**1**), 34 (**7**)
4	26	BTA21_19.0 Mb	197	3.5	18 (**1**), 86 (**5**), 7 (**6**), 26 (**7**), 16 (**11**), 5 (**18**), 7 (**20**), 22 (**21**)
4	27	BTA9_100.5 Mb	55	12.6	32 (**7**), 10 (**9**)
4	28	BTA7_93.2 Mb	268	2.6	8 (**6**), 121 (**7**), 14 (**9**), 98 (**14**), 9 (**20**)

1 = Group of the lead SNPs that were clustered together as shown on Figure 7.

2 = This SNP order refers SNPs, which are given on Table 4.

3 = 28 linear indexes corresponding to the 28 lead SNPs.

4 = Total number of significant SNPs which are significantly (*P*<10^−5^) associated with each of linear indexes.

5 = only presented if number of significant SNPs on each chromosome (*P*<10^−5^) is more than four. Chromosome number is in parentheses.

**Figure 8:**
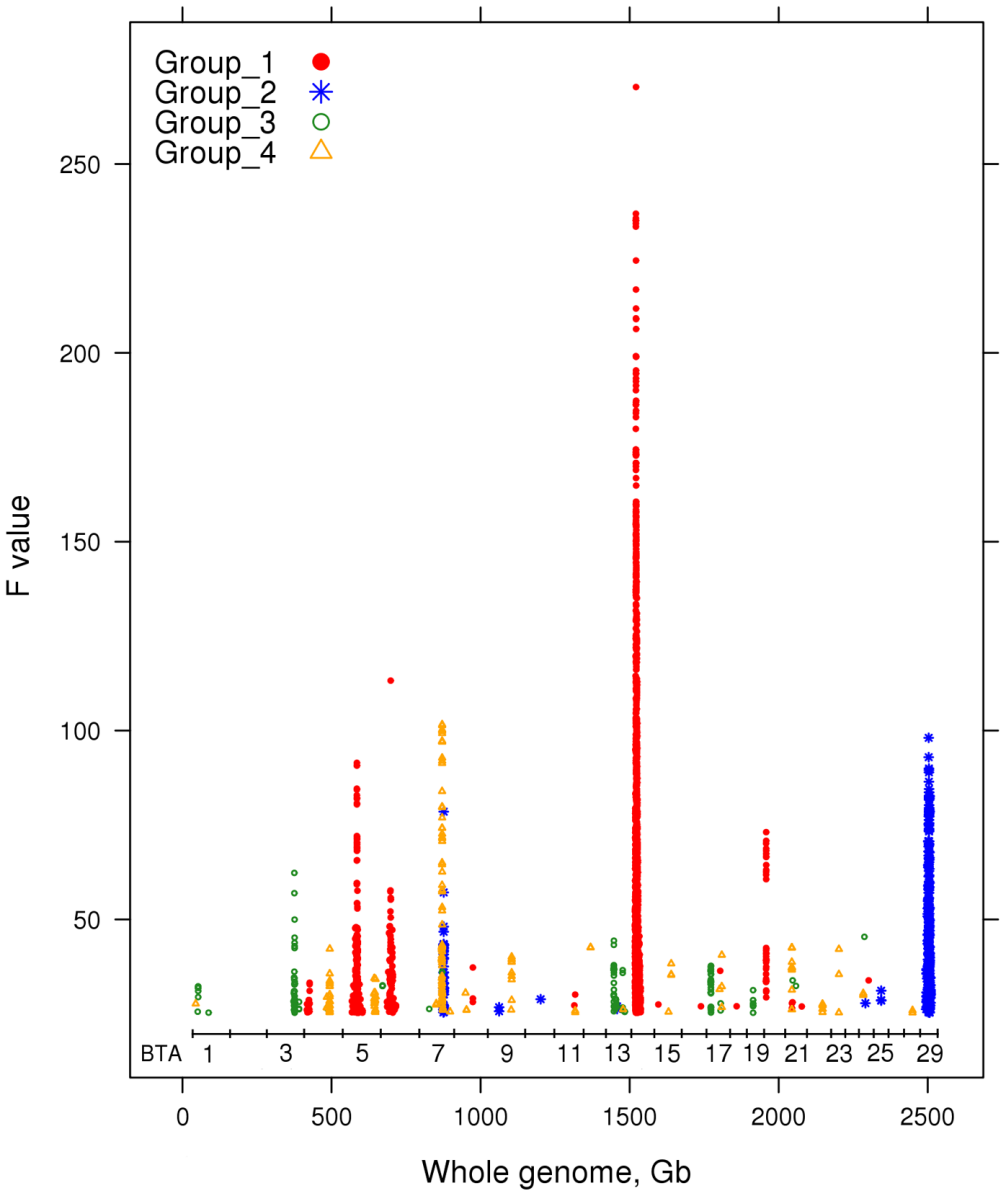
The positions of the best SNPs (5×10^−7^) that are highly correlated with each group of linear index.

The additional significant SNPs were assigned to the 4 groups as follows. For each SNP, the linear index with which it showed the most significant association (*P*<5×10^−7^) was found. The SNP was then assigned to the same group as the lead SNP defining that linear index. The results are shown in Figure 8. Usually this procedure identified a set of closely linked SNPs, presumably indicating a single QTL. Therefore we kept in the final group only the most significant SNP (*P*<5×10^−7^) from each set. The number of significant SNPs assigned to each of the 4 Groups were as follows: 1) 2,076; 2) 398; 3) 169 and 4) 176. The positions or regions of the most significant SNPs in the expanded groups are listed in Table 7.

**Table 7:** Positions of plausible candidate genes identified in *Bos taurus* that were within 1 Mb of the significant SNPs of each group.

				Plausible candidate genes			
Name of most sig. SNP	BTA	Position (bp)^1^	No. SNP^2^	Gene	Gene name	BTA	Start and end position
***Group 1***							
BovineHD0400000122	4	665622	1				
BovineHD0400000284	4	1261978	1				
BovineHD0400000645	4	2702626–2722628	3				
BovineHD0400001481	4	5191680–5447479	5	*BT.24356*	growth factor receptor-bound protein 10	4	5104899_5244348
BovineHD0400002366	4	7108902–7669014	4				
BovineHD0400002703	4	8722920–8745104	2				
BovineHD0400003043	4	9218984–9965927	5				
BovineHD0500010279	5	35255792–35896741	7	*ANO6*	anoctamin 6	5	35059697_35150452
BovineHD0500035418	5	46889976–48889976	141	*HMGA2*	High Mobility Group AT-Hook 2	5	48053846_48199963
BovineHD0500018592	5	66386971–66484917	4	*IGF-I*	insulin-like growth factor 1 (somatomedin C)	5	66532877_66604734
BovineHD0600010976	6	39093712–41093712	23				
BovineHD0800024885	8	83575973–83693221	3				
BovineHD1100030149	11	100667966–103599900	2	*GFI1B*	growth factor independent 1B transcription repressor	11	103039731_103051510
BovineHD1400007259	14	24515640–26515640	347	*PLAG1*	pleiomorphic adenoma gene 1	14	25007291_25009296
BovineHD1500003824	15	15384481	1				
BovineHD1600020726	16	72937754	1				
ARS-BFGL-NGS-36082	17	55916203	1				
BovineHD1800010790	18	35817150	1	*LCAT*	lecithin-cholesterol acyltransferase	18	35544370_35547611
BovineHD2000001543	20	4510146–4917418	40				
BovineHD2100006256	21	19315388–21252249	3	*PLIN*	perilipin	21	21502826_21516686
BovineHD2100015006	21	52213249	1				
BovineHD2500008003	25	28785015	1				
***Group 2***							
BovineHD0700028765	7	97444057–99791666	48	*CAST*	calpastatin	7	98444979_98581253
BovineHD0900015975	9	58434589–58447291	2				
BovineHD1000026655	10	92188144	1				
BovineHD1300013410	13	45909405–45911134	2				
BovineHD2500005078	25	18023277	1	*ACSM*	acyl-CoA synthetase medium-chain family members (5, 2A, 1, and 3)	25	18207129_18656582
BovineHD2600007456	26	28012143–28015564	3				
BovineHD2900012374	29	39901491–41901491	16	*DAGLA*	diacylglycerol lipase, alpha	29	40857731_40883995
				*FADS*	fatty acid desaturases (1,2, and 3)	29	40940932_41102449
BovineHD2900013185	29	43070926–45070926	98	*CAPN1*	calpain 1, (mu/I) large subunit	29	44064429_44089990
				*FIBP*	fibroblast growth factor (acidic) intracellular binding protein	29	44665294_44669337
***Group 3***							
BovineHD0100014747	1	51055086–52556409	9	*MYH15*	myosin, heavy chain 15	1	53530575_53687569
BovineHD0100024891	1	87504288	1	*ACTL6A*	actin-like 6A	1	88129517_88160918
BovineHD0300023058	3	79105316–81105316	26	*LEPR*	similar to leptin receptor; leptin receptor	3	80071689_80147000
BovineHD0300027512	3	95779023–95808937	3				
BTA-71063-no-rs	4	67772757	1				
BovineHD0600003224	6	12746688–12749442	3				
BovineHD0700014472	7	49991320	1	*BT.33253*	myotilin	7	50941047_50958425
BovineHD0700027193	7	93075709–93076407	2				
BovineHD1300010190	13	34099934–36099934	33	*ZEB1*	Zinc Finger E-Box Binding Homeobox 1	13	34063175–34261299
BovineHD1300018388	13	63952212–64544354	4	*BT.86382*	growth differentiation factor 5	13	65340132_65343889
BovineHD1300020415	13	71426238–71429231	2	*LPIN3*	lipin 3	13	70666141_70683589
BovineHD1700007016	17	24451063–24892281	37				
BovineHD1700016275	17	57243957–57313932	2	*ARPC3*	actin related protein 2/3 complex, subunit 3, 21 kDa	17	56573543_56584354
				*BT.105634*	myosin, light chain 2, regulatory, cardiac, slow	17	56953813_56961603
BovineHD1900007319	19	25043894–25097956	6	*BT.50868*	spermatogenesis associated 22	19	24809409_24824295
BovineHD2100006370	21	21632180–32790802	2	*PLIN*	perilipin	21	21502826_21516686
BovineHD2500004094	25	14547288	1	*BFAR*	bifunctional apoptosis regulator	25	13640454_13672230
				*BT.100599*	myosin, heavy chain 11, smooth muscle	25	14218281_14343745
***Group 4***							
BovineHD0100046441	1	44437965	1	*COL8A1*	collagen, type VIII, alpha 1	1	43541936_43717619
BovineHD0400018623	4	67742508–67780601	3				
BovineHD0400021462	4	77156871–77655595	23	*IGFBP3*	insulin-like growth factor binding protein 3	4	76705105_76712709
BovineHD0500030537	5	106417996–107475266	10	*FGF*	fibroblast growth factors (6 and 23)	5	106157909_106216757
BovineHD0700021658	7	72824476–73605795	2	*BT.27996*	adrenergic, alpha-1B-, receptor	7	73611117_73672127
BovineHD0700027239	7	92244933–94244933	55	*ARRDC3*	arrestin domain containing 3	7	93240419_93253094
BovineHD0700027869	7	95667222–95714161	3				
BovineHD0800002287	8	7222286	1				
BovineHD0800017674	8	59156184	1				
BovineHD0800018611	8	62416119–62419858	2				
BovineHD0900028542	9	99035883	1				
BovineHD0900029128	9	100519621–100535463	9	*PACRG*	PARK2 co-regulated	9	99649026_100180707
BovineHD1100030134	11	103536624–103761234	2	*GFI1B*	growth factor independent 1B transcription repressor	11	103039731_103051510
				*LCN9*	lipocalin 9	11	103327151_103328557
BovineHD1200013180	12	47984330–47987280	2				
BovineHD1300018707	13	65917704	1	*BT.86382*	growth differentiation factor 5	13	65340132_65343889
				*GHRH*	growth hormone releasing hormone	13	66863225_66872531
BovineHD1500014253	15	49447046	1				
BovineHD1500016882	15	58463005–58469454	3				
BovineHD1700015195	17	53758523	1				
BovineHD1700017438	17	61218439–61227950	3	*HRK*	harakiri, BCL2 interacting protein (contains only BH3 domain)	17	60417162_60437117
BovineHD2100005354	21	18979918–19055070	11				
BovineHD2200014376	22	50346158–50399252	7				
BovineHD2300012740	23	43906223–43925150	3				
BovineHD2500002877	25	10725405–10730359	3				
BovineHD4100018660	28	36064247–36104864	2				

1 For regions where several highly significant SNP were observed, the most significant SNP is presented;

2 Number of SNP that were significant at *P*<5×10^−7^ within the specified region; sig. = significant.

### Candidate genes

For each SNP or group of SNPs in Table 7 we examined the genes within 1 Mb and, in some cases, identified a plausible candidate for the phenotypic effect (Table 7). Focusing on those regions with multiple SNPs, the genes *CAPN1*, *CAST*, and *PLAG1*, were again identified, which are strongly identified with meat quality and growth in previous cattle studies [16]–[18]. In addition, we identified the genomic regions that include the *HMGA2*, *LEPR*, *DAGLA*, *ZEB1*, *IGFBP3*, *FGF6* and *ARRDC3* genes as having strong genetic effects in cattle. *HMGA2* and *LEPR* are well known to have effects on fatness and body composition in pigs [19], [20]. SNP in the promoter of *IGFBP3* have been shown to affect the level of *IGFBP3* in humans, which affects availability of circulating IGF1 and has a multitude of effects on growth and development [21]. Here we show a strong effect for *IGFBP3*, where previous results for marbling or backfat have either been small or non-significant [22], [23]. Differences in gene expression of *FGF6* has been shown to be associated to muscle development in cattle [24], and here we show that genetic variation at *FGF6* is associated with effects on Group 4 traits, which include muscling and yield traits. *ARRDC3* is a gene involved in beta adrenergic receptor regulation in cell culture [25], and beta adrenergic receptor modulation is involved in tenderness, growth and muscularity in cattle [26], [27]. Here we show that variation at *ARRDC3* is strongly associated with growth and muscularity traits in these cattle.

## Discussion

We demonstrated that our multi-trait analysis has a lower FDR than any one single trait analysis (at the same significance test *P*-value) and that these SNPs are more likely to be validated in a separate sample of animals. The most significant SNP in the multi-trait analysis provides a consensus position across the traits affected and a consistent set of estimates of the QTL for the various traits. This is in contrast to single trait analyses that often report the effect of different SNPs on each trait while neglecting the pattern of effects of the QTL across traits. For instance, the multi-trait analysis makes it clear that the QTL in Group 1 increase weight and decrease fatness whereas QTL in Group 3 increase both.

Other methods are available for multi-trait analysis [1], but the method used here has advantages. It can and has been applied to data where the individuals measured for different traits are partially overlapping and where the individual level data are not available. It utilises the estimated effects of the SNPs as well as the *P*-values and takes account of traits where the effects of a SNP may be in opposite directions. An alternative approach is illustrated by Andreasson et al. [28] in which only SNPs that are significant for one trait are tested for a second trait. However, this approach is only applicable when different individuals have been recorded for each trait and does not generalise easily to more than 2 traits.

Ideally, in the multi-trait analysis, the matrix V (the correlation matrix among the SNP effects) would contain the covariances among the errors in the estimates of SNP effects. The error variance of a t-value with 1000's of degrees of freedom is very close to 1.0. Our approximation to V also has diagonal elements of 1.0 because it is a correlation matrix. The covariance between the errors in t-values for two different traits depends on the overlap in individuals measured for the two traits. If the two traits are recorded on different individuals, there is no covariance among the errors; whereas if the two traits are measured on the same individuals, the error covariance will be mainly determined by the phenotypic correlation between the traits because single SNPs explain little of the phenotypic variance. We approximate these error covariances by the correlation between t-values across 729,068 SNPs. Since most SNPs have little association with a given trait, these correlations represent phenotypic correlations in the case where both traits are measured on the same individuals. If the two traits are measured on different individuals, then the correlation of t-values is close to zero as it should be. And if there is a partial overlap between the individuals measured for the two traits, then the correlation of t-values will represent this. Thus the meta-analysis used here, although approximate, appropriately models the variances and covariances among the t-values regardless of the overlap in individuals measured for the different traits. Therefore we hope it will be widely useful including in the analysis of published GWAS results where only the effect of each SNP and its standard error are available.

Bolormaa et al. [4] carried out a multi-trait GWAS by performing a principle component analysis of the traits and then single trait GWAS on the uncorrelated principle components. The final test statistic was a sum of the individual principle component chi-squared values. The analysis used in the current paper gives very similar results to those of Bolormaa et al. [4] but the previous method requires that individual data is available and that all individuals are measured for all traits.

We distinguished between two linked QTL and one QTL with pleiotropic effects using two types of evidence. When one QTL explains the results, the SNPs in the region are highly correlated in their effects across traits and when the best SNP is fitted in the model the significance of the effects of the other SNPs drops markedly as illustrated by the results for BTA 14 in Figure 5. Conversely, when there are two or more QTLs in a small region, such as BTA7_93-98 Mb, the SNPs show low correlations across traits and are still significant after the most significant is included in the model (Figure 6). There are few reports in the literature that aim to distinguish between linked and pleiotropic QTLs [29]–[31]. Karasik et al. [29] and Olsen et al. [30] conclude that pleiotropy exists if the same SNP or QTL region affects both traits. David et al. [31]'s method uses only two traits but, like ours, is based on the correlation between SNP effects.

Pleiotropy of individual QTL contributes to the genetic correlation between traits. If two traits have a high and positive genetic correlation it implies that most QTL affect them both in the same direction. For instance, most SNPs with significant effects affect height and weight in the same direction and thus help to explain the known high genetic correlation between these two traits [32]. Past research [33], [34] has also found a positive genetic correlation between meat tenderness and marbling or intra-muscular fat. Consistent with that we found that SNPs that increase tenderness (decrease LLPF) usually increase marbling (CMARB or CIMF; Table 5). Similarly, SNPs that increase IGF1 concentration nearly always decrease age at puberty explaining the negative genetic correlation between these traits. A low genetic correlation between two traits might imply that they are controlled by different QTL but it could also indicate some QTL affect them in the same direction and some in opposite directions. For instance, a low genetic correlation between weight and fatness [34] could be explained by the fact that some QTL affect weight and fatness in the same direction (Group 3) whereas others affect them in opposite directions (Group 1).

Some significant SNPs map near to already known genes with effects on the traits studied, such as calpain 1, calpastatin and *PLAG1*. In other cases there are candidates that are homologous to known genes affecting growth and composition in other species (e.g., *HMGA2*). However, there are QTL in Table 7 for which we could find no obvious candidate in cattle.

We defined 4 groups of SNPs by a cluster analysis of the 28 lead SNPs such that SNPs within a group have a somewhat similar pattern of effects across traits. These groups were expanded by including SNPs whose effects were correlated with those of one of the lead SNPs in the group. If the 4 groups of QTL represent different physiological pathways, one might expect the genes that map near the QTL of a group to show some similarity of function. To an extent this is so. Group 2 SNPs, which are associated with tenderness, include SNPs near calpain 1 (*CAPN1*) and calpastatin (*CAST*) that affect tenderness via muscle fibre degradation [12]–[15]. Other SNPs in group 2 are close to genes involved in fat metabolism (acyl-CoA synthetase and fatty acid desaturase). This may be coincidental but there is a known genetic correlation between intra-muscular fat and tenderness [34] and SNPs in group 2 tend to affect both traits (Table 5).

Of the SNPs in Group 1, one on BTA 14 probably tags *PLAG1*, the 2 SNPs on BTA 5 are near *HMGA2* and *IGF1*, respectively, the SNP on BTA 21 is near *PLIN*, the SNP on BTA 6 is near *CCKAR* and within 2 Mb of *NCAPG*, all of which have been reported to affect size in other species [35]–[41]. The mechanism by which they do this is uncertain. *HMGA2* is a transcription factor needed to prevent stem cells from differentiating and thus a polymorphism in it could affect growth prior to terminal differentiation. *IGF1* is the growth factor that mediates the effect of growth hormone. *PLAG1* is a transcription factor thought to regulate expression of *IGF1*, which is important in growth. *PLIN* encodes a growth factor receptor-binding protein that interacts with insulin receptors and insulin-like growth-factor 1 receptor (*IGF1R*). *PLIN* is required for maximal liposis and utilization of adipose tissue [42].

Group 3 SNPs affect fatness and the SNP on BTA 3 are in the leptin receptor gene (*LEPR*), the SNP on BTA 13 is near *LPIN3* (which regulates fatty acid metabolism), the SNP on BTA 21 is again near *PLIN* indicating that this QTL has similarities to both groups 1 and 3 (Table 7). *LEPR* is a receptor for leptin and is involved in the regulation of fat metabolism. It is known that leptin is an adipocyte-specific hormone that regulates body weight and plays a key role in regulating energy intake and expenditure. Other Group 3 SNPs were near genes that encode muscle proteins such as myosin and actin, which are involved with muscle contraction (e.g., myotilin on BTA 7 encodes a cytoskeletal protein which plays a significant role in the stability of thin filaments during muscle contraction). We do not know which, if any, of these genes contain causal mutations but it seems likely that the QTL within each group are somewhat heterogeneous. This would not be surprising given the complexity of feedback mechanisms of growth of mammals. It may be that changes to either muscle or fat growth indirectly affect growth of the other tissue.

However, even QTL that have a similar pattern of pleiotropic effects, show differences in the detail of this pattern. For instance, the Group 1 QTL might all be described as affecting ‘mature size’, but the one on BTA 14, which is presumably *PLAG1* [9], [11], has a greater effect on reproductive traits than the others in Group 2. On the other hand, the QTL on BTA 5 has an unusual pattern of effects in that it redistributes fat from the P8 site on the rump to the rib and intramuscular depots. This QTL maps close to the gene *HMGA2*, which contains polymorphisms affecting growth, fatness and fat distribution in humans, mice, horse, and pigs [35], [36], [38], [39].

Based on these results, it would appear that, although QTL can be put in meaningful groups, each QTL has its own pattern of effects. For instance, *PLAG1* might be described as a gene affecting mature size but with additional effects on reproduction, while *HMGA2* affects mature size and fat distribution. This could be explained if genes exist in a network rather than in pathways. Then each gene has a unique position in the network and therefore a unique pattern of effects. In addition, many genes occur in multiple networks in which they can have different functions.

Beef cattle breeders seek to change the genetic merit of their cattle for many of the traits studied here. The pattern of effects of each QTL indicates that some would be more useful for selection than others. Some QTL have desirable effects on one trait but undesirable effects on other traits. For instance, Brahman breeders have evidently selected for the allele of *PLAG1* that increases mature size [11], but this has decreased the fertility of their cattle. On the other hand, some QTL have an allele with desirable effects on more than one trait and appear to be good targets for selection. For instance, the QTL on BTA 4 has an allele that increases retail beef yield and marbling but also decreases sub-cutaneous fat, which is a highly valuable pattern. Selection for this allele would be beneficial in cattle intended for most markets because cattle prices reflect yield and intramuscular fat scores, whereas subcutaneous fat generally enters the by-product stream.

In conclusion, we have used a novel multi-trait, meta-analysis to map QTL with pleiotropic effects on 32 traits describing stature, growth, and reproduction. The distinctive features of the method are 1) increased power to detect and map QTL and 2) use of summary data on SNP effects when individual level data are not available. We have also presented two methods (one new) for distinguishing between linked and pleiotropic QTL (the correlation between SNP effects across traits and the effects of one SNP conditional on the effect of another SNP), and found pleiotropic QTL which appear to cluster into 4 functional groups based their trait effects. We used linear indices of 22 traits 1) to validate the effects of SNPs on multiple traits and 2) to find additional QTL belonging to the 4 functional groups. We identified candidate genes in those groups that have known biological functions consistent with the biology of the traits.

## Materials and Methods

Animal Care and Use Committee approval was not obtained for this study because no new animals were handled in this experiment. The experiment was performed on trait records and DNA samples that had been collected previously.

### SNP data

In total, 729,068 SNP data were used in this study. Those SNP were obtained from 5 different SNP panels: the Illumina HD Bovine SNP chip (http://res.illumina.com/documents/products/datasheets/datasheet_bovinehd.pdf) comprising 777,963 SNP markers; the BovineSNP50K version 1 and version 2 BeadChip (Illumina, San Diego) comprising 54,001 and 54,609 SNP, respectively; the IlluminaSNP7K panel comprising 6,909 SNP; and the ParalleleSNP10K chip (Affymetrix, Santa Clara, CA) comprising 11,932 SNP. All SNP were mapped to the UMD 3.1 build of the bovine genome sequence assembled by the Centre for Bioinformatics and Computational Biology at University of Maryland (CBCB) (http://www.cbcb.umd.edu/research/bos_taurus_assembly.shtml. High density SNP genotypes were imputed for all animals using Beagle (Browning and Browning, 2011). The approaches used for performing quality control and imputation were described in [8]. The details of the quality control and imputation were recapitulated below.

Stringent quality control procedures were applied to the SNP data of each platform. SNP were excluded if the call rate per SNP (this is the proportion of SNP genotypes that have a GC (Illumina GenCall) score above 0.6) was less than 90% or they had duplicate map positions (two SNP with the same position but with different names) or an extreme departure from Hardy-Weinberg equilibrium (e.g., SNP in autosomal chromosomes with both homozygous genotypes observed, but no heterozygotes). Furthermore, if the call rate per individual was less than 90%, those animals were removed from the SNP data. The SNP data were edited within breed group and within each platform and were subsequently combined.

After all the quality control tests were applied, 729,068 SNP of the HD SNP chip were retained on 1,698 animals and the missing genotypes were filled using the BEAGLE program [43]. Imputation was done using 30 iterations of BEAGLE. The genotypes for each SNP were encoded in the top/top Illumina A/B format and then genotypes were reduced to 0, 1, and 2 copies of the B allele. The imputations of the 7 K, 10 K and 50 K SNP genotype data to the 729 068 SNPs were performed in two sequential stages: from 7 K or 10 K or 50 K data to a common 50 K data set and then from the common 50 K data set to 800 K data. In the first stage imputation was done within breed, using 30 iterations of Beagle. In the second stage, the HD genotypes of each breed type (501 *B. taurus* and 520 *B. indicus*) were used as a reference set to impute from the 50 K genotypes of each pure breed within the corresponding breed type. For the four composite breeds, all the HD genotypes (1,698) were used as a reference set to impute the 50 K genotypes of each composite breed up to 800 K. The number of genotypes for each platform used as reference animals for imputation and number of animals used in this study is given in Table 8. The mean R^2^ values, for the accuracy of imputation provided by BEAGLE, are in Table 9. After imputation, an additional quality control step was applied based on comparing allele frequencies between SNP platforms to detect SNP with very different allele frequencies indicating incorrect conversion between platforms. In total, 10,191 animals, which had a record for at least one trait and also had SNP genotypes, were used in this study.

**Table 8:** The number of genotypes used by each breed within each platform.^1^

Breed name	Breed type	HD^2^	50 K^2^	HD^3^	50 K^3^	7 K^3^	10 K^3^
Angus	Bt	194	1080	133	1016	282	312
Murray Gray	Bt	36	100	19	97	107	
Hereford	Bt	74	498	30	437	146	
Shorthorn	Bt	123	639	30	594	93	
Charolais	Bt	13					
Limousin	Bt	61					
Brahman	Bi	520	4124	303	2706	380	
Belmont Red	Bt×Bi	97	404		265	258	
Santa Gertrudis	Bt×Bi	166	1041	45	213	340	
Tropical Composites	Bt×Bi	369	1073	322	1073	464	
Droughtmaster	Bt×Bi	45					
recent Brahman crosses	Bt×Bi		462		455	72	
Total number		1698	9421	882	6856	2141	312

1 = Columns 6–8 are sourced from [8].

2 = number of HD (high density SNP chip) and 50 K genotypes used for imputation as reference animals;

3 = number of animals with HD, 50 K, 7 K, and 10 K genotypes used in this study; Bt = *Bos taurus*; Bi = *Bos indicus*; Bt×Bi = composite breed.

**Table 9:** The accuracy of imputation (R^2^) obtained from Beagle of the genotyped data.^1^

Imputation	7 K data		50 K data		10 K data	
/breed^2^	7 K to 50 K	50 K to 800 K	50 K to 50 K	50 K to 800 K	3 K to 50 K	50 K to 800 K
AAMG	0.89	0.94	0.98	0.95	0.88	0.96
BB	0.78	0.90	0.96	0.90		
BR	0.80	0.92	0.98	0.93		
BX			0.95	0.85		
HH	0.75	0.92	0.97	0.90		
SG	0.75	0.93	0.94	0.93		
SS	0.87	0.92	0.96	0.91		
TC	0.76	0.93	0.96	0.95		
Mean	0.80	0.92	0.97	0.90	0.88	0.96

1 = this table is sourced from [8];

2 = Angus (AA), Brahman (BB), Belmont Red (BR), Hereford (HH), recent Brahman crosses (BX), Murray Grey (MG), Santa Gertrudis (SG), Shorthorn (SS) and Tropical Composites (TC); AAMG = genotypes of AA and MG animals were imputed together.

### Animals and phenotypes

The cattle were sourced from 9 different populations of 3 breed types. They include 4 different *Bos taurus* (Bt) breeds (Angus, Murray Grey, Shorthorn, Hereford), 1 *Bos indicus* (Bi) breed (Brahman cattle), 3 composite (Bt×Bi) breeds (Belmont Red, Santa Gertrudis, Tropical composites), and 1 recent Brahman cross population (F1 crosses of Brahman with Limousin, Charolais, Angus, Shorthorn, and Hereford). Details on population structure of those animals have previously been described by Bolormaa et al. [8].

Phenotypes for 32 different traits including growth, feed intake, carcass, meat quality, and reproduction traits were collated from 5 different sources: The data sources included the Beef Co-operative Research Centre Phase I (CRCI), Phase II (CRCII), Phase III (CRCIII), the Trangie selection lines, the Durham Shorthorn group (the detailed description is reported by Bolormaa et al. [8] and Zhang et al. [44]. Not all cattle were measured for all traits. The trait definitions, number of records for each trait and heritability estimate and mean and its SD of each trait are shown in Table 1.

### Single-trait GWAS

Mixed models fitting fixed and random effects simultaneously were used for estimating heritabilities and associations with SNP. Variances of random effects were estimated in each case by REML. The estimates of heritability were calculated based on all animals with phenotype and genotype data and their 5-generation-ancestors using the following mixed model: trait ∼ mean + fixed effects + animal + error; with animal and error fitted as random effects. The individual animal data for the 32 traits were used to perform genome wide association studies (GWAS), in which each SNP was tested for an association with the trait. The association between each SNP and each of the traits was assessed by a regression analysis using the ASReml software [45]. The model used was the same as for estimating heritability, but SNP_i_ (SNP_i_, i = 1, 2, 3, … , 729068) was additionally fitted as a covariate one at time (trait ∼ mean + fixed effects + SNP_i_ + animal + error). The model used to analyse the traits consistently included dataset, breed, cohort and sex as fixed effects. Other fixed effects varied by trait. The fixed effects were fitted as nested within a dataset. Further details of the models used in the analysis are reported by Johnston et al. [46], Reverter et al. [47], Robinson and Oddy [48], Barwick et al. [49], Wolcott et al. [50], Bolormaa et al. [8], and Zhang et al. [44].

### Multi-trait meta-analysis chi-squared statistic

We applied a new statistic to find the significance level of SNPs in a multi-trait analysis. This statistic determines the importance of the effects of SNP_i_ (i = 1, 2, 3, …, 729068) across all (32) traits studied. Our multi-trait test statistic is approximately distributed as a chi-squared with 32 degrees of freedom. It tests a null hypothesis stating that the SNP does not affect any of the traits. For each SNP, the multi-trait statistic was calculated by the formula:
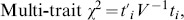
where ***t_i_*** is a 32×1 vector of the signed t-values of SNP**_i_** for the 32 traits ***t_i_′*** is a transpose of vector ***t_i_*** (1×32) ***V^−1^*** is an inverse of the 32×32 correlation matrix where the correlation between two traits is the correlation over the 729,068 estimated SNP effects (signed t-values) of the two traits.

This approximation method is justified as follows: t-values based on many degrees of freedom have an error variance close to 1.0 and t^2^ is distributed as a *χ* ^2^_(1)_ under the null hypothesis. Therefore, if the SNP effects on n different traits were estimated independently with no error covariance, the sum of the t^2^ (i.e., 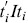, where *I* is an identity matrix) would be distributed as a chi-squared with n degrees of freedom. Our approximate analysis would generate exactly this test statistic if the t values for different traits had no error covariance. If the t values for different traits had an error (co)variance matrix *D*, then the correct test statistic would be 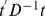 distributed as a chi-squared with n degrees of freedom. We approximate *D* by the correlation between the estimated SNP effects across the 729,068 SNPs. We assume that most SNPs have little or no effect on most traits, so most of the (co)variance between effects is error covariance. However, the SNPs that do have a real effect on a trait will inflate the variance of SNP effects above 1.0. Therefore we convert the covariance matrix of SNP effects (*D*) to a correlation matrix (*V*) because this returns the diagonal elements to 1.0 which we know is the correct error variance for t statistics. Although it is not proof of the method, perhaps we offer the following intuitive analysis. If the SNP effects on different traits were estimated in independent GWAS then the correlation of SNP effects would be low and *V*≈*I* and the test statistic would be the sum of independent chi-squares, as expected. On the other hand, if the SNP effects on different traits were estimated from the same individuals, then the correlation of error variances would be driven mainly by the phenotypic correlations between the traits. In this case the correlation of SNP effects would also reflect these phenotypic correlations and the test statistic we use would be a good approximation of the correct test statistic.

### Power of multi-trait meta-analysis to detect QTL

#### False discovery rate (FDR)

The increase of power of QTL detection was investigated by comparing FDR calculated in multi-trait test with FDR calculated in single-trait GWAS. Following Bolormaa et al. [51], the false discovery rate was calculated as 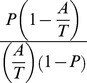 where *P* is the *P*-value tested (e.g., 0.00001), *A* is the number of SNP that were significant at the *P* -value tested and *T* is the total number of SNP tested.

#### Validation of SNP effects

To validate SNP effects in the multi-trait test, we developed a new approach that uses a linear index of traits that had maximum correlation with the SNP. This new approach was carried out as follows: 1) Splitting data as reference and validation populations; 2) Predicting missing phenotypes using multiple regression approach; 3) Performing single-trait GWAS in the reference population to get the SNP effects based on only the reference population; 4) Calculating a linear index of 22 traits for each SNP, which had maximum association with the SNP in reference population; and 5) Validating SNP effects using GWAS to discover if there is any association between the corresponding linear index and SNP.

1. *Splitting data*. For validation purposes, a 5-fold cross validation schema was carried out. The whole data were split into five sets by allocating all of the offspring of randomly selected sires to one of the five datasets. Then one of the 5 divisions was used as a validation population and the other 4 divisions as the reference population. In this way no animal used for validation had paternal half sibs in the reference population.
2. *Predicting missing phenotypes*. The linear index on individual animals could only be calculated for animals with all traits measured. This required individual animal level data. Therefore this process was restricted to the 22 non-reproduction traits since the cows and bulls, on which the reproductive traits were measured, were not recorded for carcass traits. Even among these 22 traits, not all animals were measured for all traits. Before the missing phenotypes were predicted, the raw phenotypes for each trait were corrected for fixed effects using the following model: corrected phenotype = phenotype – fixed effects. So missing values were filled in by a prediction using multiple regression on the traits that were recorded on that animal. This multiple regression procedure uses the actual effects (not signed t values) of 729,068 SNPs for 22 traits that were estimated based on all animals (reference and validation population) in order to have the same units with phenotype values. For each animal, SNP effects for the 22 traits were reordered so that those traits with a phenotypic value preceded those traits with missing values. Then the (co)variance matrix of SNP effects among the 22 traits were calculated and inverted: 
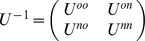
, where *U^oo^*is the inverse of (co)variance matrix of SNP effects between the traits with a phenotype value, *U^nn^* is the inverse of (co)variance matrix SNP effects between traits with a missing record, and *U^on^*vs *U^no^*is the inverse of (co)variance matrix of the traits with and without a missing record. The missing phenotypes (*y_n_*) were then predicted using the following formula: 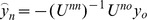, where *y_o_* is a vector of the traits measured on a particular animal.
3. *GWAS in reference population*. The individual trait GWAS and the multi-trait significance test on signed t-values described in the previous sections were performed using only the reference population. Only the most significant SNP from a sliding window of every 1-Mb-interval was retained to avoid identifying a large number of closely linked SNPs whose association with traits is due to the same QTL or due to LD to an index SNP. If a SNP in each 1-Mb-interval was significant at *P*<10^−5^ then it was selected to be validated in the validation population using the linear index of 22 traits.
4. *Calculating linear index*. A linear index (*y_I_*) of 22 traits that has maximum correlation in the reference population with each selected SNP was derived. This linear index was calculated for each animal. The phenotype values and the effects of the SNP are used to calculate the linear index, so the actual effects of the SNP (not signed t values) were in the same units as the trait values. The following formula was used to calculate a linear index: 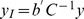, where *b′* is the transpose of a vector of the estimated effects of the SNP on the 22 traits (1×22) that was estimated from only the reference population, *C^−1^* is an inverse of the 22×22 (co)variance matrix among the 22 traits calculated from the estimated SNP effects of 729,068 SNPs only in the reference population, and y is a 22×1 vector of the phenotype values for 22 traits for each animal in the validation sample.
5. *Validating SNP effects using GWAS*. The association between each linear index (*y_I_*) and each SNP was then tested in the validation population. The *y_I_* was treated as a new trait (dependent variable). The association was assessed by a regression analysis (GWAS) using the following model: *y_I_*∼ mean + SNP_i_ + animal + error, where animal and error were fitted as random effects and SNP_i_ were fitted as a covariate one at a time (other fixed effects were removed from the trait measurements before forming the linear index).

In order to see whether the SNPs validated in the validation population have the same direction of effects (positive or negative) as SNPs in the reference population, we repeated the steps 2, 4, and 5 by using the phenotypes of the reference population instead of the phenotypes of the validation population. Then the directions of SNP effects for the linear index in both reference and validation populations were checked and the proportion of SNPs whose effects were in the same direction in the reference population was calculated.

### Multi-trait meta-analysis tends to find SNPs near genes

The gene start and stop positions were identified using Ensembl (www.ensembl.org/biomart/) and SNPs were classified according to their distance from the nearest gene. The SNPs were placed in bins 1) <100 kb upstream of the start site or downstream of the stop site, 2) 100–200 kb upstream or downstream, etc., in 100 kb bins. SNPs between the start and stop sites were placed in a separate bin (called 0 kb from the nearest gene). For each bin the proportion of SNPs that were significant (*P*<10^−5^) in the multi-trait analysis was divided by the total number of SNPs in that bin.

### Single-trait GWAS to test pleiotropy or linkage

The SNP effects estimated from single-trait GWAS based on all animals were used to investigate the relationships between SNPs. For each pair of SNPs, the correlation of the effects across 32 traits was calculated. Highly positive or negative correlations indicate 2 SNPs with the same pattern of effects across traits.

### Conditional analysis to test pleiotropy or linkage

The 28 lead SNPs were selected as follows: On each chromosome the one or two most significant SNPs (*P*<10^−5^), based on the multi-trait analysis, were selected. Two SNPs on the same chromosome were only selected if they clearly represented two different QTL based on the test for pleiotropy vs linkage. In no case were the SNPs less than 2 Mb apart.

The regression analyses (GWAS) were performed again but additionally the 28 lead SNPs were fitted simultaneously in the model. The statistical model used was trait ∼ mean + fixed effects + SNP_i_ + leadSNP_1_ + leadSNP_2_ + leadSNP_3_ + … + leadSNP_28_ + animal + error; with animal and error fitted as random effects. The *i*th SNP (SNP_i_, i = 1, 2, 3, … , 729068) and 28 lead SNPs were fitted simultaneously as covariate effects. Then a multi-trait chi-squared statistic was calculated for each SNP to test the effects of the SNP across traits after fitting the 28 lead SNPs.

### Cluster analysis

For each pair of SNPs among the 28 lead SNPs, the correlation of their effects across the 32 traits was calculated. Then this correlation matrix was used to do the hierarchical clustering of the 28 lead SNPs leading to 4 groups or clusters as shown in the dendrogram drawn using the heatmap function of the R program [52].

### Finding additional SNPs in the 4 groups defined by the cluster analysis

For each of the 28 lead SNPs, we searched for additional SNPs with a similar pattern of effects. To do this we used the linear index that showed the highest association with a lead SNP, as previously defined for validation of the multi-trait analysis. A new GWAS was performed for each of 28 linear indexes (*y_I_*) treating it as a new trait (dependent variable). The following model was used: *y_I_* ∼ mean + fixed effects + SNP_i_ + animal + error, where animal and error were fitted as random effects and the *i*th SNP (SNP_i_, i = 1, 2, 3, … , 729068) was fitted as a covariate effect.

The SNPs that have significant associations (*P*<5×10^−7^) with at least one of the indexes based on lead SNPs were selected for assigning into 4 groups. These additional significant SNPs were assigned to the same group as the lead SNP whose linear index with which they had the most significant association.

### Annotating SNPs

The genes that occur within 1 Mb of the SNPs in this expanded list were identified using Ensembl (www.ensembl.org/biomart/) and, in some cases, a plausible candidate for the phenotypic effect was identified.

## References

[1] Solovieff N, Cotsapas C, Lee PH, Purcell SM, Smoller JW (2013) Pleiotropy in complex traits: challenges and strategies. Nat Rev Genet 14: 483–495. 23752797 10.1038/nrg3461 PMC4104202

[2] Korol AB, Ronin YI, Itskovich AM, Peng J, Nevo E (2001) Enhanced efficiency of quantitative trait loci mapping analysis based on multivariate complexes of quantitative traits. Genetics 157: 1789–1803. 11290731 10.1093/genetics/157.4.1789 PMC1461583

[3] Gilbert H, Leroy P (2007) Methods for the detection of multiple linked QTL applied to a mixture of full and half sib families. Genet Sel Evol 39: 139–158. 17306198 10.1186/1297-9686-39-2-139 PMC2682834

[4] Bolormaa S, Pryce JE, Hayes BJ, Goddard ME (2010) Multivariate analysis of a genome-wide association study in dairy cattle. J Dairy Sci 93: 3818–3833. 20655452 10.3168/jds.2009-2980

[5] Yang J, Lee T, Kim J, Cho M-Ch, Han B-G, et al (2013) Ubiquitous polygenicity of human complex traits: Genome-wide analysis of 49 traits in Koreans. PLoS Genetics 9: e1003355. 23505390 10.1371/journal.pgen.1003355 PMC3591292

[6] Korol AB, Ronin YI, Itskovich AM, Peng J, Nevo E (2001) Enhanced efficiency of quantitative trait loci mapping analysis based on multivariate complexes of quantitative traits. Genetics 157: 1789–1803. 11290731 10.1093/genetics/157.4.1789 PMC1461583

[7] Knott SA, Haley CS (2000) Multitrait least squares for quantitative trait loci detection. Genetics 156: 899–911. 11014835 10.1093/genetics/156.2.899 PMC1461263

[8] Bolormaa S, Pryce JE, Kemper K, Savin K, Hayes BJ, et al (2013) Accuracy of prediction of genomic breeding values for residual feed intake, carcass and meat quality traits in Bos taurus, Bos indicus and composite beef cattle. J Anim Sci 91: 3088–3104. 23658330 10.2527/jas.2012-5827

[9] Karim L, Takeda H, Lin L, Druet T, Arias JAC, et al (2011) Variants modulating the expression of a chromosome domain encompassing PLAG1 influence bovine stature. Nat Genet 43: 405–413. 21516082 10.1038/ng.814

[10] Hawken RJ, Zhang YD, Fortes MRS, Collis E, Barris WC, et al (2011) Genome-wide association studies of female reproduction in tropically adapted beef cattle. J Anim Sci 90: 1398–1410. 22100599 10.2527/jas.2011-4410

[11] Fortes MRS, Kemper K, Sasazaki S, Reverter A, Pryce JE, et al (2013) Evidence for pleiotropism and recent selection in the PLAG1 region in Australian Beef cattle. Anim Genet 44: 636–47 doi:10.1111/age.12075 23909810 10.1111/age.12075

[12] Barendse WJ (2002) inventor; Commonwealth Scientific and Industrial Research Organisation, The State of Queensland through its Department of Primary Industries, The University of New England, The State of New South Wales through its Department of Agriculture, and Meat and Livestock Australia Limited, assignees (2002) DNA markers for meat tenderness. International Patent Application WO 02064820

[13] Barendse W, Harrison BE, Hawken RJ, Ferguson DM, Thompson JM, et al (2007) Epistasis between calpain 1 and its inhibitor calpastatin within breeds of cattle. Genetics 176: 2601–2610. 17603104 10.1534/genetics.107.074328 PMC1950658

[14] Page BT, Casas E, Heaton MP, Cullen NG, Hyndman DL, et al (2002) Evaluation of single nucleotide polymorphisms in CAPN1 for associations with meat tenderness in cattle. J Anim Sci 80: 3077–3085. 12542147 10.2527/2002.80123077x

[15] White SN, Casas E, Wheeler TL, Shackelford SD, Koohmaraie M, et al (2005) A new single nucleotide polymorphism in CAPN1 extends the current tenderness marker test to include cattle of Bos indicus, Bos taurus, and crossbred descent. J Anim Sci 83: 2001–2008. 16100054 10.2527/2005.8392001x

[16] Karim L, Takeda H, Lin L, Druet T, Arias JAC, et al (2011) Variants modulating the expression of a chromosome domain encompassing PLAG1 influence bovine stature. Nature Genetics 43: 405–+. 21516082 10.1038/ng.814

[17] Barendse W, Harrison BE, Hawken RJ, Ferguson DM, Thompson JM, et al (2007) Epistasis between calpain 1 and its inhibitor calpastatin within breeds of cattle. Genetics 176: 2601–2610. 17603104 10.1534/genetics.107.074328 PMC1950658

[18] White SN, Casas E, Wheeler TL, Shackelford SD, Koohmaraie M, et al (2005) A new single nucleotide polymorphism in CAPN1 extends the current tenderness marker test to include cattle of *Bos indicus*, *Bos taurus*, and crossbred descent. Journal of Animal Science 83: 2001–2008. 16100054 10.2527/2005.8392001x

[19] Óvilo C, Fernández A, Noguera JL, Barragán C, Letón R, et al (2005) Fine mapping of porcine chromosome 6 QTL and LEPR effects on body composition in multiple generations of an Iberian by Landrace intercross. Genetical Research 85: 57–67. 16089036 10.1017/s0016672305007330

[20] Wimmers K, Murani E, Pas M, Chang KC, Davoli R, et al (2007) Associations of functional candidate genes derived from gene-expression profiles of prenatal porcine muscle tissue with meat quality and muscle deposition. Animal Genetics 38: 474–484. 17697135 10.1111/j.1365-2052.2007.01639.x

[21] Deal C, Ma J, Wilkin F, Paquette J, Rozen F, et al (2001) Novel promoter polymorphism in insulin-like growth factor-binding protein-3: Correlation with serum levels and interaction with known regulators. Journal of Clinical Endocrinology & Metabolism 86: 1274–1280. 11238520 10.1210/jcem.86.3.7280

[22] Cheong HS, Yoon DH, Kim LH, Park BL, Lee HW, et al (2008) Association analysis between insulin-like growth factor binding protein 3 (IGFBP3) polymorphisms and carcass traits in cattle. Asian-Australasian Journal of Animal Sciences 21: 309–313.

[23] Veneroni GB, Meirelles SL, Grossi DA, Gasparin G, Ibelli AMG, et al (2010) Prospecting candidate SNPs for backfat in Canchim beef cattle. Genetics and Molecular Research 9: 1997–2003. 20957603 10.4238/vol9-4gmr788

[24] Bernard C, Cassar-Malek I, Renand G, Hocquette JF (2009) Changes in muscle gene expression related to metabolism according to growth potential in young bulls. Meat Science 82: 205–212. 20416758 10.1016/j.meatsci.2009.01.012

[25] Nabhan JF, Pan H, Lu QA (2010) Arrestin domain-containing protein 3 recruits the NEDD4 E3 ligase to mediate ubiquitination of the beta 2-adrenergic receptor. Embo Reports 11: 605–611. 20559325 10.1038/embor.2010.80 PMC2920442

[26] Hunter RA, Sillence MN, Gazzola C, Spiers WG (1993) INCREASING ANNUAL GROWTH-RATES OF CATTLE BY REDUCING MAINTENANCE ENERGY-REQUIREMENTS. Australian Journal of Agricultural Research 44: 579–595.

[27] Warner RD, Ferguson DM, Cottrell JJ, Knee BW (2007) Acute stress induced by the preslaughter use of electric prodders causes tougher beef meat. Australian Journal of Experimental Agriculture 47: 782–788.

[28] Andreassen OA, Thompson WK, Schork AJ, Ripke S, Mattingsdal M, et al (2013) Improved detection of common variants associated with schizophrenia and bipolar disorder using pleiotropy-informed conditional false discovery rate. PLoS Genet 9 4: e1003455 doi:1003410.1001371/journal.pgen.1003455 23637625 10.1371/journal.pgen.1003455 PMC3636100

[29] Karasik D, Hsu Y, Zhou Y, Cupples LA, Kiel DP, et al (2010) Genome-wide pleiotropy of osteoporosis-related phemotypes. The Framingham study. J Bone Min Res 25: 1555–1563. 10.1002/jbmr.38 PMC3153998 20200953

[30] Olsen HG, Hayes BJ, Kent MP, Nome T, Svendsen M, et al (2011) Genome-wide association mapping in Norwegian Red cattle identifies quantitative trait loci for fertility and milk production on BTA12. Anim Genet 42: 466–474. 21906098 10.1111/j.1365-2052.2011.02179.x

[31] David I, Elsen J-M, Concordet D (2013) CLIP Test: a new fast, simple and powerful method to distinguish between linked or pleiotropic quantitative trait loci in linkage disequilibria analysis. Heredity 110: 232–238. 23250009 10.1038/hdy.2012.70 PMC3668649

[32] Vargas CA, Elzo MA, Chase CC Jr, Olson TA (2000) Genetic parameters and relationships between hip height and weight in Brahman. J Anim Sci 78: 3045–3052. 11132818 10.2527/2000.78123045x

[33] O'Connor SF, Tatum JD, Wulf DM, Green RD, GC S (1997) Genetic effects on beef tenderness in Bos indicus composite and Bos taurus cattle. J Anim Sci 75: 1822–1830. 9222838 10.2527/1997.7571822x

[34] Reverter A, Johnston DJ, Ferguson DM, Perry D, Goddard ME, et al (2003) Genetic and phenotypic characterisation of animal, carcass, and meat quality traits from temperate and tropically adapted beef breeds. 4. Correlations among animal, carcass, and meat quality traits. Aus J Agri Res 54: 149–158.

[35] Anand A, Chada K (2000) In vivo modulation of Hmgicreduces obesity. Nat Genet 24: 377–380. 10742101 10.1038/74207

[36] Kim KS, Lee JJ, Shin HY, Choi BH, Lee CK, et al (2006) Association of melanocortin 4 receptor (MC4R) and high mobility group AT-hook 1 (HMGA1) polymorphisms with pig growth and fat deposition traits. Anim Genet 37: 419–421. 16879362 10.1111/j.1365-2052.2006.01482.x

[37] Setoguchi K, Furuta M, Hirano T, Nagao T, Watanabe T, et al (2009) Cross-breed comparisons identified a critical 591-kb region for bovine carcass weight QTL (CW-2) on chromosome 6 and the Ile-442-Met substitution in NCAPG as a positional candidate. BMC Genetics 10: 43. 19653884 10.1186/1471-2156-10-43 PMC2736976

[38] Voight BF, Scott LJ, Steinthorsdottir V, Morris AP, Dina C, et al (2010) Twelve type 2 diabetes susceptibility loci identified through large-scale association analysis. Nat Genet 42: 579–589. 20581827 10.1038/ng.609 PMC3080658

[39] Makvandi-Nejad S, Hoffman GE, Allen JJ, Chu E, Gu E, et al (2012) Four Loci explain 83% of size variation in the horse. PLoS ONE 7: e39929. 22808074 10.1371/journal.pone.0039929 PMC3394777

[40] Tetens J, Widmann P, Kühn C, Thaller G (2013) A genome-wide association study indicates LCORL/NCAPG as a candidate locus for withers height in German Warmblood horses. Anim Genet 44: 467–471. 23418885 10.1111/age.12031

[41] Setoguchi K, Watanabe T, Weikard R, Albrecht E, Kühn C, Kinoshita A, Sugimoto Y, Takasuga A (2011) The SNP c.1326T>G in the non-SMC condensin I complex, subunit G (NCAPG) gene encoding a p.Ile442Met variant is associated with an increase in body frame size at puberty in cattle. Anim Genet 42: 650–655. 22035007 10.1111/j.1365-2052.2011.02196.x

[42] Tansey J, Sztalryd C, Gruia-Gray J, Roush DL, Zee JV, et al (2001) Perilipin ablation results in a lean mouse with aberrant adipocyte lipolysis, enhanced leptin production, and resistance to diet-induced obesity. PNAS 98: 6494–6499. 11371650 10.1073/pnas.101042998 PMC33496

[43] Browning BL, Browning SR (2011) A fast, powerful method for detecting identity by descent. Am J Hum Gen 88: 173–182. 10.1016/j.ajhg.2011.01.010 PMC3035716 21310274

[44] Zhang YD, Johnston DJ, Bolormaa S, Hawken RJ, Tier B (2013) Genomic selection for female reproduction in Australian tropically adapted beef cattle. Anim Prod Sci http://dx.doi.org/10.1071/AN13016.

[45] Gilmour A, Gogel BJ, Cullis BR, Thompson R (2009) ASReml User Guide Release 3.0 VSN International Ltd, Hemel Hempstead. HP1 1ES, UK.

[46] Johnston DJ, Reverter A, Ferguson DM, Thompson JM, Burrow HM (2003) Genetic and phenotypic characterisation of animal, carcass and meat quality traits for temperate and tropically adapted beef breeds. 3. Meat quality traits. Aust J Agr Res 54: 135–147.

[47] Reverter A, Johnston DJ, Perry D, Goddard ME, Burrow HM (2003) Genetic and phenotypic characterisation of animal, carcass and meat quality traits for temperate and tropically adapted beef breeds. 2. Abattoir carcass traits. Aust J Agric Res 54: 119–134.

[48] Robinson DL, Oddy VH (2004) Genetic parameters for feed efficiency, fatness, muscle area and feeding behaviour of feedlot finished beef cattle. Livest Prod Sci 90: 255–270.

[49] Barwick SA, Wolcott ML, Johnston DJ, Burrow HM, MT S (2009) Genetics of steer daily feed intake and residual feed intake in tropical beef genotypes and relationships among intake, body composition, growth and other post-weaning measures. Anim Prod Sci 49: 351–366.

[50] Wolcott ML, Johnston DJ, Barwick SA, Iker CL, Thompson JM, et al (2009) Genetics of meat quality and carcass traits and the impact of tenderstretching in two tropical beef genotypes. Anim Prod Sci 49: 383–398.

[51] Bolormaa S, Hayes BJ, Savin K, Hawkin R, Barendse W, et al (2011) Genome wide association studies for feedlot and growth traits in cattle. J Anim Sci 89: 1684–1697. 21239664 10.2527/jas.2010-3079

[52] The R Core Team (2013) R: A Language and Environment for Statistical Computing. Version 3.0.1.

